# METTL protein family: focusing on the occurrence, progression and treatment of cancer

**DOI:** 10.1186/s40364-024-00652-3

**Published:** 2024-09-17

**Authors:** Huhu Zhang, Fulin Sun, Shuyao Jiang, Fanghao Yang, Xiaolei Dong, Guoxiang Liu, Mengjun Wang, Ya Li, Mohan Su, Ziyuan Wen, Chunjuan Yu, Chenkai Fan, Xiaoxia Li, Zhe Zhang, Lina Yang, Bing Li

**Affiliations:** 1 Department of Genetics and Cell Biology, School of Basic Medicine, Qingdao University, Qingdao, 266071 China; 2 Health Science Center, Qingdao University, Qingdao, 266071 China; 3 Department of Clinical Laboratory, Weifang People’s Hospital, 151, Guangwen Streer, Weifang, 261041 China; 4 Department of Dermatology, The Affiliated Haici Hospital of Qingdao University, Qingdao, 266003 China

**Keywords:** METTL protein family, Cancer progression, Biomarker, Treatment, Inhibitor

## Abstract

Methyltransferase-like protein is a ubiquitous enzyme-like protein in the human body, with binding domains for nucleic acids, proteins and other small molecules, and plays an important role in a variety of biological behaviours in normal organisms and diseases, characterised by the presence of a methyltransferase-like structural domain and a structurally conserved SAM-binding domain formed by the seven-stranded β-fold structure in the center of the protein. With the deepening of research, the METTL protein family has been found to be abnormally expressed in a variety of tumor diseases, and the clarification of its relationship with tumor diseases can be used as a molecular therapeutic target and has an important role in the prognosis of tumors. In this paper, we review the structure, biological process, immunotherapy, drug-targeted therapy, and markers of the METTL protein family to provide new ideas for the diagnosis and treatment of tumors.

**Supplementary Information:**

The online version contains supplementary material available at 10.1186/s40364-024-00652-3.

## Introduction

Methyltransferase catalyzes the transfer of methyl to DNA, RNA, proteins and other biomolecules [1]. Methyltransferase-like (METTL) proteins are a seven-chain methyltransferase family with an S-adenosylmethionine binding domain [2]. METTL is a protein encoded by a highly conserved polygenic family and is widely found in animals and plants [3, 4]. Thirty-four members of the METTL protein family have been found in mammals, twelve of which modify DNA or RNA, fifteen modify protein residues, and six whose functions are currently unknown [5]. They are uniquely expressed in specific tissues and organs; for example, the expression level of METTL3 was significantly higher in the roots, stems, leaves, garland, petals, stamens, pistil and calyx of cotton [6]. METTL14 expression is significantly reduced in patients with retinitis pigmentosa (RP) [7]. METTL23 is closely related to Intellectual disability (ID) and fully segregates with the phenotype of ID and facial dysmorphism [8].

METTL proteins also have a broad substrate range and different species of METTL proteins are involved in different cellular biological processes, including cardiovascular disease, ophthalmic diseases, tumor progression and viral replication [9]. Impaired m [7]G tRNA modification upon METTL1 depletion resulted in decreased cell proliferation capacities of lung cancer [10]. METTL3 overexpressing mice exhibited significant myocardial hypertonicity but no accelerated dysfunction during pressure overload stress [11]. Replication of SARS-CoV-2, the agent responsible for the COVID-19 pandemic, and a seasonal human β-coronavirus HCoV-OC43, can be suppressed by depletion of METTL3 or cytoplasmic m6A reader proteins YTHDF1 and YTHDF3 [12]. The METTL protein family also plays a function in cancer progression, and seventeen members of the METTL protein family have been successively shown to have pro- or anti-cancer effects [13]. These include METTL1-9, METTL11, METTL13-18, METTL21 and METTL24. Tissue-specific METTL proteins exist in different cancers, including bladder cancer [14], hepatocellular carcinoma [15], breast cancer [16] and colorectal cancer [9]. The METTL protein family affects a variety of signaling pathways and can be used as markers of tumor development. In addition, some METTL proteins are potential therapeutic targets for tumors. To explore the effects of these drugs and their inhibitors can provide better strategies for early diagnosis, clinical treatment and prognosis of tumors.

## The molecular structure of METTL protein family

**Fig. 1:**
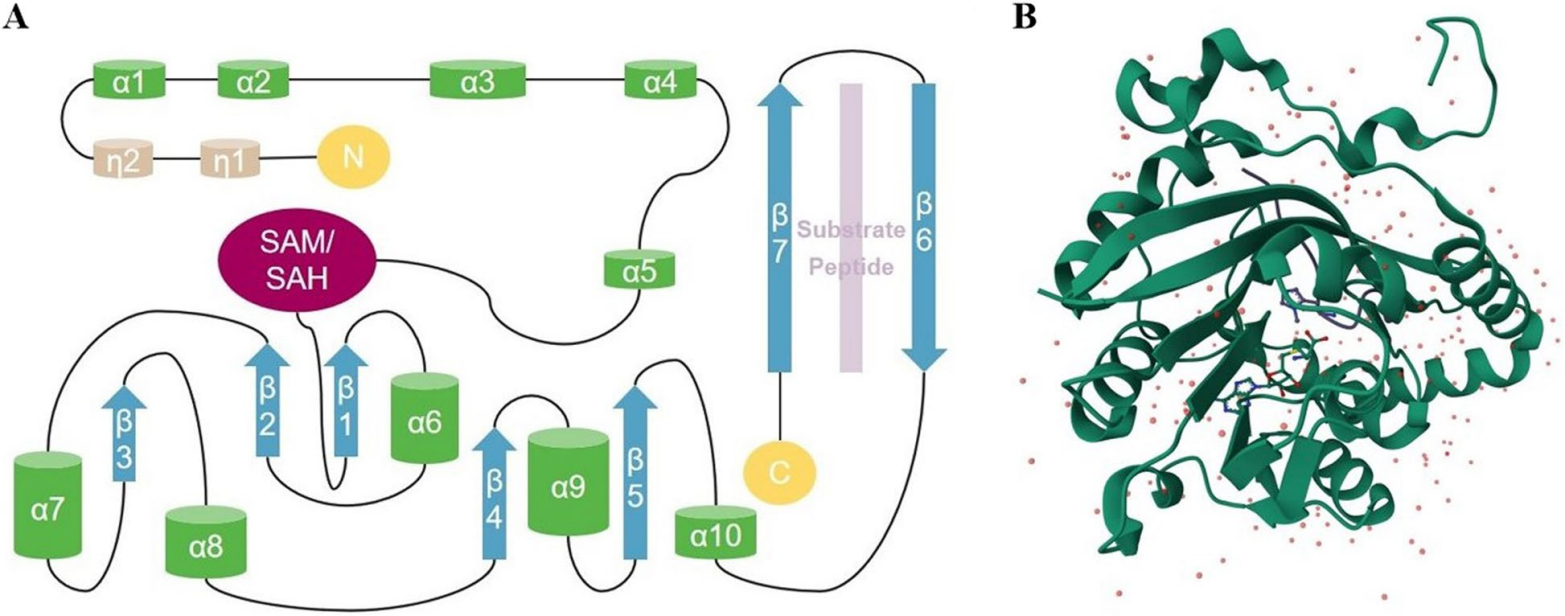
The molecular structure of METTL protein. METTL protein family is a subfamily of seven β-folded chain methyltransferases in the order β3β2β1β4β5β7β6. METTL protein family consists of an N-terminal RNA-binding structural domain, a methyltransferase domain (MTD), and a C-terminal VCR-conserved structural domain. Figure **A** is the figure structure of METTL9, figure **B** is the three-dimensional structure of METTL9, corresponding to figure **A**

The METTL protein family is a subfamily of seven β-folded chain methyltransferases in the order β3β2β1β4β5β7β6 [17]. The METTL protein family consists of an N-terminal RNA-binding structural domain, a methyltransferase domain (MTD), and a C-terminal VCR-conserved structural domain [18]. The RNA-binding structural domain consists of approximately the first 40 or so amino acids of the N-terminal end, and includes multiple positively charged amino acid residues [19]. In terms of spatial structure, these amino acid residues together form a groove for accommodating RNA that can anchor the target RNA and provide spatial conditions for its methyl transfer [20]. The positively charged amino acid residues at the entrance form a “pocket” structure that facilitates the binding and methylation of the target RNA by the METTL protein [21](Fig. 1).

Members of the METTL family have a conserved S-adenosylmethionine (SAM) binding structural domain that methylates not only proteins, but also nucleotides and other small molecule metabolites [22, 23]. The methyltransferase structural domain contains an ordered polypeptide loop, an NPPF motif, and a disordered loop. The ordered polypeptide loop is an equilibrium sensor for intracellular SAM molecules [18, 24]. Under different SAM concentrations, the ordered polypeptide ring will change its own conformation and regulate the methylation modification efficiency of METTL proteins [25].The NPPF motif, as the active site of MTD, can prompt the substrate RNA to bind with SAM at the pocket and induce the substrate RNA to undergo methylation [26]. The charge state of the disordered loop can regulate the binding of METTL protein to RNA, which in turn affects the methylation of the target RNA [23]. When the negatively charged amino acid residues of the disordered ring are mutated to positively charged amino acid residues, the methyl modification function of the METTL protein remains unchanged [23]. However, when some of the positively charged amino acid residues of the loop were mutated to negatively charged, the binding ability of the METTL protein to RNA was reduced, which might be related to the charge repulsion of RNA [27]. The above three functional structures regulate METTL protein methyltransferase activity in their own unique ways [28].

The VCR structural domain, also known as the conserved region, consists of VCR1, VCR2 and a disordered region [28]. At present, the function and mechanism of the VCR structural domain have not been fully elucidated [29]. Some studies have shown that the VCR structural domain can regulate the splicing of RNA and the methylation process of its target RNAs, but it is worthwhile to further explore whether the VCR structural domain also has other functions [23].

## The role of METTL protein family in cancer progression

**Fig. 2:**
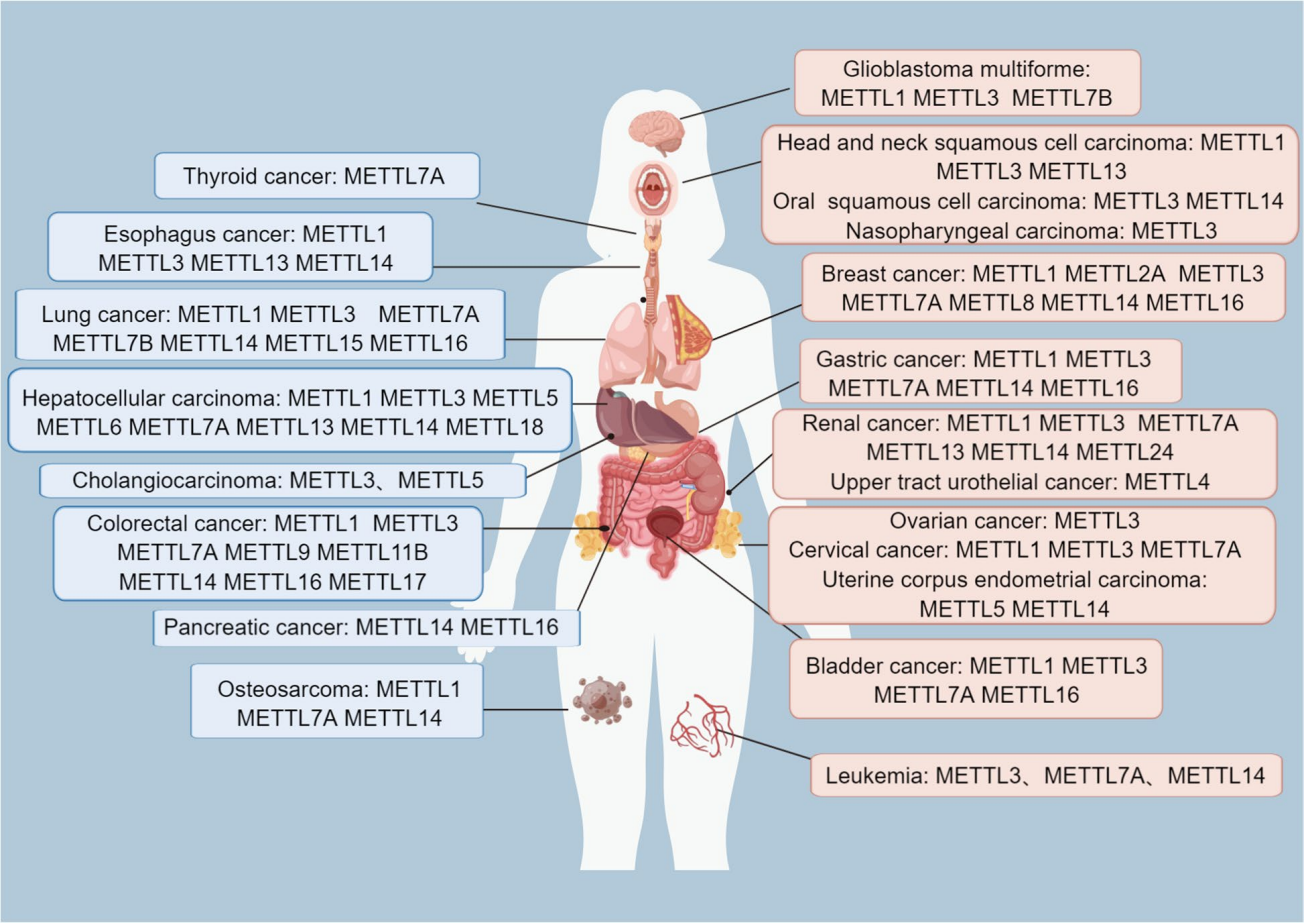
The distribution of METTL protein family members in different cancers

The biological process of cancer includes the cascade reaction process of cancer cells escaping from the primary site, penetrating blood vessels, circulating dissemination, and distal colonization [30, 31]. Existing evidence shows that METTL protein family regulates tumor cell proliferation, cell cycle, invasion, migration, autophagy and apoptosis through signaling pathways. In addition, members of the METTL protein family have different expression patterns in different cancers (Fig. 2). Although members of the METTL protein family are conserved in structure, they play different regulatory roles in tumorigenetic development, respectively promoting or inhibiting the tumorigenetic development (Table 1).

### The role of METTL protein family in cell proliferation

The METTL protein family can promote or inhibit tumor proliferation by affecting cell membrane, cytoskeleton formation and signaling pathway [17]. In osteosarcoma, METTL1 is highly expressed in osteosarcoma and correlates with poor patient prognosis. Knockdown of METTL1 results in reduced levels of tRNA m7G modification and impairs osteosarcoma proliferation in vitro and in vivo [32]. METTL1 is highly expressed in nasopharyngeal carcinoma, and METTL1 promotes the proliferation of nasopharyngeal carcinoma cells by upregulating the Wnt/β-catenin signaling pathway [33]. METTL3 knockdown significantly inhibited the growth of gastric cancer(GC), and METTL3 acted as a methyltransferase to regulate the expression of genes related to the TGF-β/smad pathway, especially METTL3 modifies Smad3 mRNA through m6A [34]. Similarly, in bladder cancer, METTL3 exerts oncogenic effects by interacting with DGCR8 and positively regulating the pri-miR221/222 process in an m6a-dependent manner [35]. METTL4 is highly expressed in upper tract urothelial cancer and METTL4 induces nuclear 6 mA modification during tumor metastasis [36]. METTL5 regulates hepatocellular carcinoma (HCC) proliferation through numerous pro-oncogenic USP5-c-Myc signaling cascades [37]. METTL6 catalyzes the formation of 3-methylcytidine at the C32 site of a specific serine tRNA heteroreceptor, and deletion of METTL6 leads to changes in ribosome occupancy and RNA levels, as well as impaired pluripotency in HCC [38]. METTL13 promoted GC cell growth through an eEF1A/HN1L positive feedback loop [39]. In addition, METTL13 also promoted HCC cell proliferation by upregulating TCF3 and ZEB1 [40].

Conversely, METTL14 is downregulated in GC, and METTL14 overexpression inhibits the PI3K/AKT/mTOR pathway to suppress GC proliferation [41]. METTL14 is downregulated in bladder cancer tissues. METTL14 also inhibits the proliferation of bladder cancer cells [42]. Knockout of METTL14 decreased the m6A level of lnc RNA XIST, reduced the recognition and degradation of XIST by YTHDF2, and promoted the expression of XIST and the proliferative capacity of colorectal cancer [43]. METTL16 activates the p21 signalling pathway by m6A modification and inhibits the proliferation of pancreatic ductal adenocarcinoma cells [44]. In addition, METTL16 can inhibit the translation of DVL2 mRNA through m6A modification, thereby regulating the Wnt/β-catenin signalling pathway and inhibiting the proliferation of pancreatic ductal adenocarcinoma cells [45]. In summary, the METTL protein family regulates tumor proliferation by targeting the stability of the internal structure of RNA methylation as well as multiple signaling pathways in a wide range of malignant tumors, including HCC and GC, and different members of the METTL protein family can play opposite roles in the same tumor.

### The role of METTL protein family in cell cycle

Cell cycle regulation is a cornerstone of tumor development. Methylation modifications directly influence tumor cell growth dynamics by precisely regulating RNA behaviour [46]. The METTL protein family plays critical roles in cell cycle regulation in a complex manner. METTL1 knockdown suppressed colorectal cancer growth and G_1_/S phase transition and interacted with checkpoint kinase 2 and suppress its protein expression [47]. In osteosarcoma, knockdown of METTL3 decreases mRNA and protein levels of DRG1, which further leads to G_2_/M phase cell cycle arrest and inhibits osteosarcoma cell proliferation [48]. METTL7B expression is elevated in lung cancer, and deletion of METTL7B significantly reduces cyclin D1, a key regulator of the G_1_/S transition, leading to G_0_/G_1_ arrest and inhibition of lung cancer proliferation [49]. METTL16 upregulates cyclin D1 expression by enhancing cyclin D1 mRNA stability through m6A modification, which in turn accelerates the G_1_/S phase transition to promote gastric cancer cell proliferation [50]. Elevated METTL18 expression level could activate the G_2_/M checkpoint, KRAS signaling pathway and mitotic spindle, and knockdown of METTL18 significantly inhibited the proliferation of HCC cells [51]. The METTL protein family plays an important role in biological processes such as cytoskeletal reorganization as well as cell cycle regulation by methylating relevant substrates itself and interacting with multiple signaling pathways. As cell cycle regulators, the METTL family of proteins has only just begun to be studied.

### The role of METTL protein family in invasion and metastasis

**Fig. 3:**
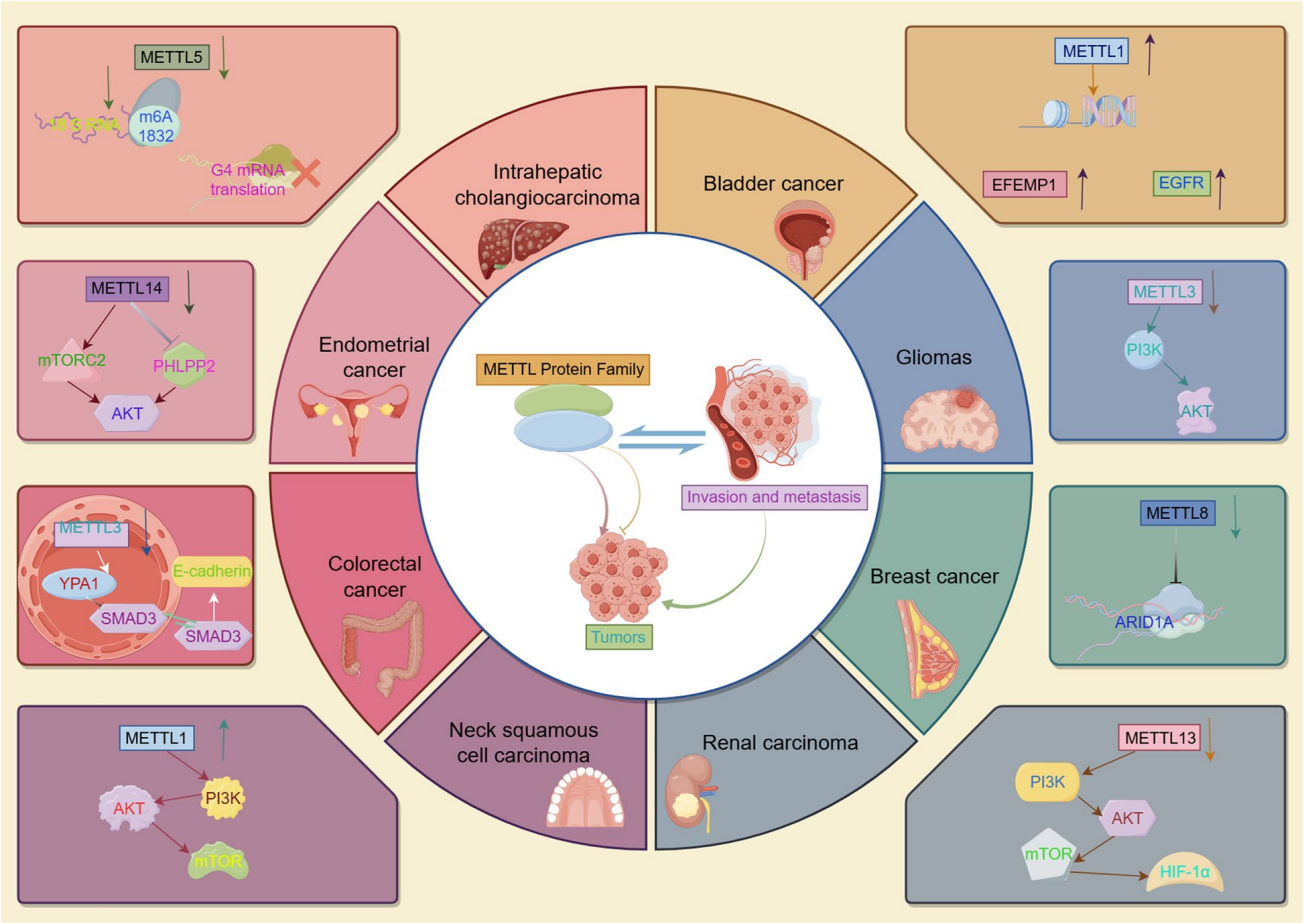
Signaling pathways of METTL protein family regulating tumor cell invasion and metastasis. The METTL protein family has a dual role in cancer proliferation. METTL1, METTL5, and METTL14 promote cancer proliferation, and conversely, METTL3, METTL8 and METTL13 inhibit cancer proliferation

Numerous studies have confirmed that the most common cause of poor clinical outcomes in malignant tumors is recurrence and metastasis, and the vast majority of tumors undergo EMT during tumor progression, allowing tumor cells to acquire infiltrative and metastatic properties during progression, accelerating the formation of malignant tumors and allowing for increased invasive metastatic capacity [46, 52]. Increasing evidence has elucidated the vital role of the METTL protein family in cancer invasion and metastasis. METTL protein family regulates cancer invasion and metastasis in multiple ways (Fig. 3). First, METTL protein family regulates EMT to promote tumor invasion and metastasis. METTL1 was highly expressed in bladder cancer, and its level was correlated with poor patient prognosis. Silencing METTL1 suppresses the migration and invasion of bladder cancer cells in vitro and in vivo [14]. In gliomas, the expression of METTL3 was significantly downregulated in tumor tissues compared to adjacent normal tissues. Down-regulation of METTL3 enhances the ability of the PI3K/AKT pathway to promote glioma migration and invasion [53]. In breast cancer, METTL3 inhibits E-cadherin expression through m6A modification of EZH2, thereby inducing EMT to promote tumor invasion and metastasis [54]. METTL8 expression is elevated in breast cancer, and knockdown of METTL8 inhibits tumor cell growth and strongly blocks tumor cell migration [55]. METTL13 is lowly expressed in renal cell carcinoma, and knockdown of METTL13 promotes the PI3K/AKT/mTOR/HIF-1α pathway leading to migratory ability and invasiveness of renal cell carcinoma cells as well as elevated Vimentin and N-cadherin [56]. Second, the METTL protein family regulates angiogenesis to promote tumor invasion and metastasis. In bladder cancer, METTL3 deletion resulted in reduced m6A abundance in specific regions of tyrosine kinase endothelial (TEK) and VEGF-A mRNA and decreased levels of TEK and VEGF-A mRNA and protein [57].

Finally, the METTL protein family regulates tumor invasion and metastasis by mediating methylation modifications. METTL1 knockout markedly reduced the proliferation, migration, and invasive ability of head and neck squamous cell carcinoma cells, and by decreasing the level of m7G tRNA modification inhibited oncogenic gene translation and PI3K/AKT/mTOR signaling pathway [58]. In colorectal cancer, METTL3 promotes the expression of circ1662 by m6A modification of circ1662 through binding to its flanking strand, while circ1662 can regulate SMAD3 expression by accelerating the nuclear localization of YAP1, which ultimately promotes invasive metastasis of colorectal cancer [59]. Deletion of METTL5 affects the modification of 18 S rRNA m6A, which hinders ribosome synthesis and inhibits translation of G-tetraploid-rich mRNAs in the transforming growth factor TGF-β pathway, inhibiting intrahepatic cholangiocarcinoma invasion and migration [60]. The expression of circ-METTL9 was significantly up-regulated in colorectal cancer tissues and significantly elevated in patients with advanced colorectal cancer tumors. Overexpression of circ-METTL9 promotes colorectal cancer cell proliferation and migration in vitro, as well as colorectal cancer tumor growth and metastasis in vivo [9]. METTL14 leads to a decrease in m6A methylation and activation of the AKT pathway, which promotes the proliferation and migration of endometrial cancer cells. The increase in AKT activity is dependent on a decrease in PHLPP2 expression and an increase in mTORC2 expression. METTL14 leads to a decrease in m6A methylation and activation of the AKT pathway, which promotes the proliferation and migration of endometrial cancer cells. The increase in AKT activity is dependent on a decrease in PHLPP2 expression and an increase in mTORC2 expression [61]. METTL16 reduced the m6A methylation level of iron-dead GPX4 mRNA and significantly promoted the degradation of GPX4 mRNA, suggesting that METTL16 enhances the expression level of GPX4 through m6A modification, and thus inhibits iron-death to promote the proliferation of breast cancer cells [62]. In colorectal cancer, METTL17 inhibition disrupts mitochondrial function, energy metabolism, and enhances intracellular and mitochondrial lipid peroxidation and ROS levels. In addition, METTL17 inhibition significantly reduced mitochondrial RNA methylation, including m4C, m5C, m3C, m7G, and m6A, resulting in impaired translation of mitochondrial protein-coding genes [63]. Although the role of METTL proteins in tumor development has been extensively studied, the molecular mechanism of their regulation of tumor invasion and metastasis still needs to be further clarified. Elucidating the molecular mechanisms of METTL proteins in tumor invasion and metastasis will be beneficial to tumor therapy.

### The role of METTL protein family in cell apoptosis

The METTL family of proteins is closely related to tumors, and its function in promoting or inhibiting apoptosis has led to its importance in the diagnosis and treatment of malignant tumors. METTL1 is significantly upregulated in advanced neuroblastoma, knockdown of METTL1 would result in a significant increase in apoptosis of neuroblastoma cells [64]. METTL3 mediated the elevation of COL10A1 expression, promoted its mRNA m6A modification, promoted ROS content, and inhibited the production of SOD and GPX, thereby accelerating the growth and inhibiting the apoptosis of lung squamous cell carcinoma [65]. METTL5 expression is elevated in uterine corpus endometrial carcinoma, and uterine corpus endometrial carcinoma patients with high METTL5 expression have a poorer prognosis. Knockdown of METTL5 decreased CEA, CA199, CA153, HE4 protein levels and increased apoptosis [66]. The expression of METTL14 was higher in pancreatic cancer tissues than in non-tumor tissues, and knockdown of METT14 promoted apoptosis by reducing the expression levels of AMPKα, ERK1/2 [67]. METTL15 expression is up-regulated in lung cancer tissues and cells. METTL15 silencing inhibits lung cancer cell proliferation, colony formation, invasion, immune escape and promotes apoptosis [68]. In lung cancer, METTL16 deficiency prevents GCN2 protein synthesis, leading to reduced expression of ATF4 in a GCN2- eIF2α-dependent manner. Reduced ATF4 further reduces expression of the apoptosis receptor protein DR5. Meanwhile, METTL16 deficiency directly impeded the synthesis of FADD and DR5 proteins, thereby impairing apoptosis and promoting cancer cell survival [69]. Increasing understanding of the regulation of complex signaling pathways within the METTL family of proteins in tumors, particularly apoptosis, allows for more rational design of anti-cancer strategies.

### The role of METTL protein family in cell autophagy

Autophagy is lysosome-mediated cellular self-digestion, which constitutes a cyclic dynamic pathway of damaged organelles and long-lived proteins, and plays a major role in the body’s internal environmental homeostasis and tumorigenic mechanisms to ensure that cells have access to sustainably utilized energy under stress conditions such as nutrient deficiencies and the tumor microenvironment, and in this way maintain homeostasis and viability in the body [70]. In small cell lung cancer, METTL3 induces m6A methylation of DCP2, leading to DCP2 degradation and promoting mitochondrial autophagy through the Pink1-Parkin pathway [71]. METTL14 is up-regulated in pancreatic cancer and down-regulation of METTL14 improves autophagy through mTOR signaling-dependent pathways [67]. METTL14 enhances RB1CC1 expression after transcription in an m6A-IGF2BP2-dependent manner, thereby affecting autophagy and progression of oral squamous cell carcinoma. In vitro, knockdown of METTL14 significantly inhibited autophagy and promoted malignant progression, and in vivo promoted tumor growth and metastasis [72]. METTL16 significantly inhibits bladder cancer cell proliferation through the HIF-2α-METTL16- PMEPA1 autophagy axis in an m6A manner [73]. The METTL protein family will surely bring new hope for cancer treatment by regulating the powerful role of autophagy in tumor formation, growth and metastasis, giving it the regenerative ability to inhibit tumor growth.

**Table 1:** The role of the METTL protein family in cancer progression

Member	Cancer type	Type cell	Expression patterns	Effect	Ref
METTL1	Osteosarcoma	Hos and 143B cells	Protein	METTL1 is elevated in osteosarcoma and correlated with poor prognosis	[32]
	Nasopharyngeal carcinoma	NP-69, 5–8 F and 6-10B cells	mRNA and Protein	Promoting the proliferation of nasopharyngeal carcinoma	[33]
	Colorectal cancer	HCT116 and RKO cells	Protein	METTL1 knockdown suppressed G_1_/S phase transition	[47]
	Bladder cancer	5637, J82, T24 and UM-UC-3 cells	mRNA and Protein	Inhibiting the migration and invasion of bladder cancer	[14]
	Head and neck squamous cell carcinoma	UM1, SCC9 and SCC25	mRNA and Protein	Inhibiting glioma migration and invasion	[58]
	Neuroblastoma	BE2C	Protein	knockdown of METTL1 would result in a significant increase in apoptosis of neuroblastoma	[64]
METTL3	Gastric cancer	GES-1, MGC803, MNK45, and SGC7901 cells	mRNA and Protein	METTL3 knockdown significantly inhibited the growth of GC	[34]
	Bladder cancer	EJ and T24 cells	mRNA and Protein	Promoting the proliferation of bladder cancer	[35]
	Osteosarcoma	Saos-2, U2OS, MG63 and 143B cells	mRNA and Protein	Leading to G2/M phase cell cycle arrest	[48]
	Gliomas	KS-1	mRNA and Protein	Down-regulation of METTL3 promotes glioma migration and invasion	[53]
	Breast cancer	MCF10A, MCF-7 and BT474	mRNA and Protein	Promoting tumor invasion and metastasis	[54]
	Colorectal cancer	HCT116 and SW480 cells	mRNA and Protein	Promoting invasion and metastasis of colorectal cancer	[59]
	Lung squamous cell carcinoma	SW900 and LOU-NH91 cells	Protein	Inhibiting the apoptosis of lung squamous cell carcinoma	[65]
	Small cell lung cancer	H69 and H69AR cells	mRNA and Protein	METTL3 promoting mitochondrial autophagy	[71]
METTL4	Upper tract urothelial cancer	KTCC28M cell	mRNA and Protein	METTL4 is highly expressed in upper tract urothelial cancer	[36]
METTL5	Hepatocellular carcinoma	Huh7, HCCLM3, SNU182, Hep3B, and HepG2 cells	mRNA and Protein	Promoting the proliferation of HCC	[37]
	Intrahepatic cholangiocarcinoma	HCCC-9810 and QBC-939 cells	mRNA and Protein	Promoting the invasion and metastasis of ICC	[60]
	Uterine corpus endometrial carcinoma	KLE cells, RL952 cells, Ishikawa and ECC-1 cells	Protein	Knockdown of METTL5 increased apoptosis in uterine corpus endometrial carcinoma	[66]
METTL6	Hepatocellular carcinoma	HepG2 cell	mRNA	Knockout of METTL6 inhibits the proliferation ability of HCC	[38]
METTL7B	Lung cancer	A549 and PC-9	mRNA and Protein	Knockdown of METTL7B leading to G_0_/G_1_ arrest and inhibition of lung cancer proliferation	[49]
METTL8	Breast cancer	MDA-MB-231 and MCF-7	mRNA and Protein	Promoting tumor cell growth	[55]
METTL9	Colorectal cancer	Caco2, HCT116, SW480, SW620, RKO, LoVo, DLD-1 and HT-29 cell	mRNA and Protein	Overexpression of circ-METTL9 promotes colorectal cancer cell proliferation and migration	[9]
METTL13	Gastric cancer	AGS, MKN28 and MGC803 cells	Protein	Promoting GC cell proliferation	[39]
	Hepatocellular carcinoma	HCCLM6, BEL-7402 and QGY-7703 cells	Protein	Promoting HCC cell proliferation	[40]
	Renal cell carcinoma	786-O, 769-P, OS-RC-2, Caki-1 and ACHN cells	mRNA and Protein	knockdown of METTL13 promotes Renal cell carcinoma migration	[56]
METTL14	Gastric cancer	SGC-7901	mRNA and Protein	Suppressing GC proliferation	[41]
	Bladder cancer	U5637 and T24T cells	mRNA and Protein	Inhibiting the proliferation of bladder cancer	[42]
	Colorectal cancer	SW480, SW620, HCT116, LoVo and HT29 cells	mRNA and Protein	Knockout of METTL14 promoted the proliferative capacity of colorectal cancer	[43]
	Endometrial cancer	HEC-1-A	mRNA and Protein	Promoting the proliferation and migration of endometrial cancer	[61]
	Pancreatic cancer	PANC-1 and CFPAC-1 cells	mRNA and Protein	Promoting apoptosis of pancreatic cancer	[67]
	Oral squamous cell carcinoma	CAL33 and HSC3 cells	mRNA and Protein	Knockdown of METTL14 inhibits autophagy	[72]
METTL15	Lung cancer	H1299	Protein	Promoting apoptosis of lung cancer	[68]
METTL16	Pancreatic ductal adenocarcinoma	PANC-1, SW-1990, BxPC-3 and AsPC-1 cells	mRNA and Protein	Inhibiting the proliferation of pancreatic ductal adenocarcinoma	[44, 45]
	Gastric cancer	AGS, MGC803, SNU719, HGC27, SGC7901 and MKN28	mRNA and Protein	Accelerating the G_1_/S phase transition to promote gastric cancer proliferation	[50]
	Breast cancer	MCF-10 A, MDA-MB-231, MDA-MB-468, MDA-MB-453 and MCF-7 cells	mRNA and Protein	Promoting breast cancer cell proliferation by inhibiting iron death	[62]
	Lung cancer	A549 and H1299 cells	mRNA and Protein	METTL16 deficiency impairs apoptosis and promotes cancer cell survival	[69]
	Bladder cancer	T24, RT4, UMUC3, TCC, J82, 253 J and BIU87	Protein	Inhibiting bladder cancer cell proliferation	[73]
METTL17	Colorectal cancer	SW620	mRNA and Protein	Inhibition of METTL17 disrupts mitochondrial function and energy metabolism	[63]
METTL18	Hepatocellular carcinoma	HepG2, M97H, LM3, Bel7402, SK-HEP1 and Huh7 cells	Protein	Activating the G2/M checkpoint	[51]

### The role of METTL protein family in tumor immunity

The tumor microenvironment (TME) is a heterogeneous ecosystem that includes non-cellular components such as cancer cells, immune cells, stromal cells, and extracellular matrix, all of which play key roles in tumorigenesis and progression as well as in modulating the tumor’s response to immunotherapies [74–76]. The TME has been found to have the dual potential to inhibit or promote cancer. During the early stages of tumor growth, infiltrating immune cells and associated stromal components are recruited and activated by tumor cells to establish anti-tumor TME and prevent tumor formation and progression [76–78]. However, sustained immune stimulation induces depletion or remodeling of effector cells, ultimately leading to immunosuppressive TME [79]. Therefore, eliminating the suppressive effects and restoring the innate anti-tumor capacity of the immune system represent promising strategies for cancer therapy [74].

**Fig. 4:**
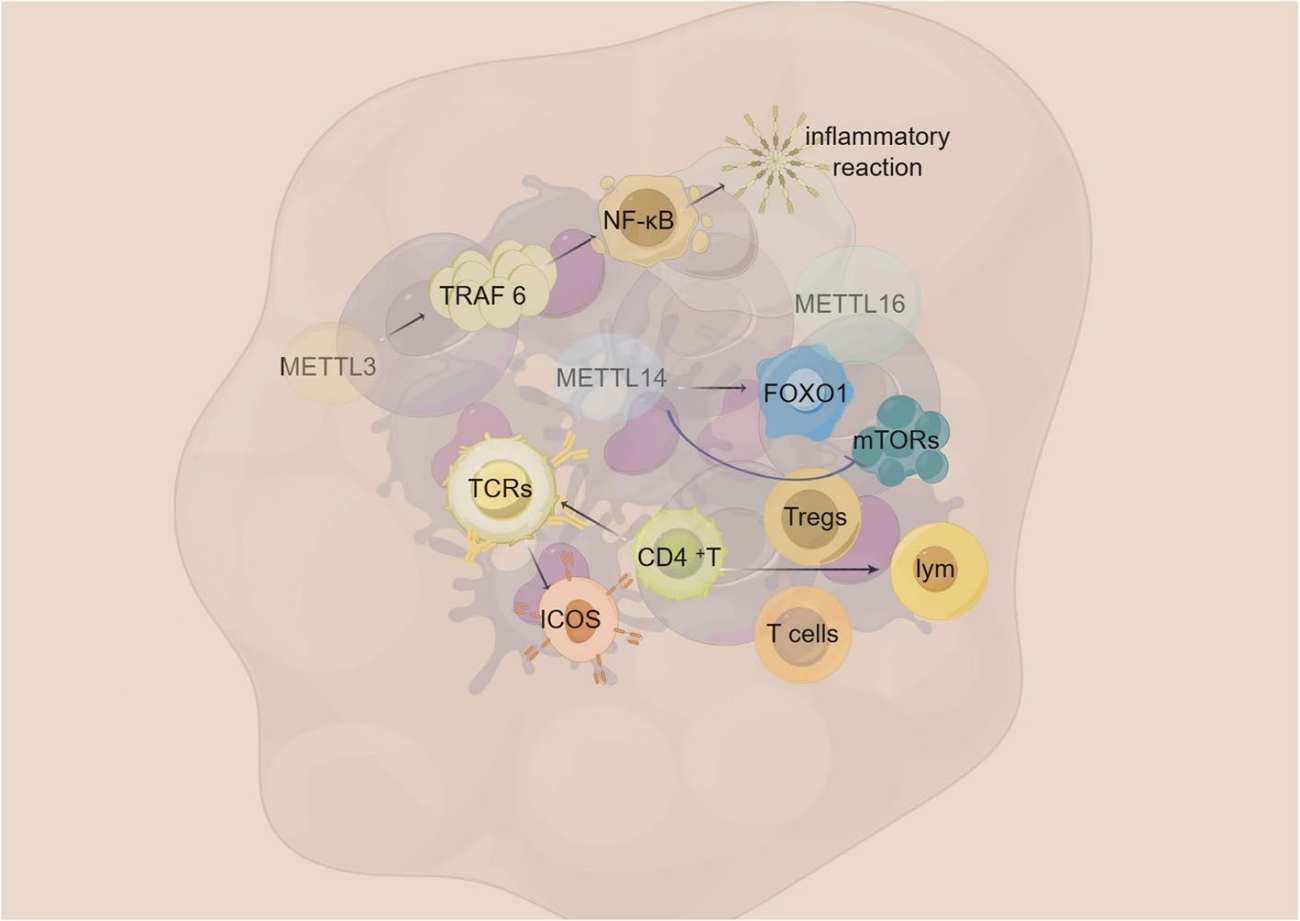
METTL protein family-associated tumor immune microenvironment. METTL3 can positively regulate the activation of the NF-κB pathway by TRAF 6 and METTL14 promotes the process of FOXO1 methylation, which in turn triggers an inflammatory response. METTL16 was found to inhibit antiviral immune response through m6A methylation modification. T-cell activation is regulated by a variety of immunomodulatory proteins. ICOS and CD40L are translocated to the surface of CD4^+^T cells. METTL3 regulate the expression of CD40L in human CD4^+^ lymphocytes and modulate CD4^+^ lymphocyte activation. Tregs are heterogeneous subpopulations of Th cells, which can be classified into tTreg as well as pTreg by constitutive expression of the transcription factor FoxP3 and play an important role in immune homeostasis by suppressing Teff

Currently, the link between epitranscriptomic abnormalities and cancer immune escape is gradually being revealed. Targeting RNA epigenetic dysregulation for therapeutic purposes can reprogram TME to improve cancer immunotherapy [80]. The METTL protein family regulates phenomena such as m6A modification of mRNAs as well as the m7G of tRNAs, which play important roles in RNA epigenetic processes. Numerous studies have confirmed that the METTL protein family plays a regulatory role in a variety of immune-related pathways. METTL3 can positively regulate the activation of the NF-κB pathway by TRAF 6, which in turn triggers an LPS-mediated inflammatory response [81]. METTL14 promotes the process of FOXO1 methylation, which in turn induces endothelial inflammation [82]. METTL16, which is evolutionarily conserved, has been found to play a major role in the small yellow croaker (a scleractinian fish) was found to inhibit antiviral immune response through m6A methylation modification [83]. Meanwhile, the METTL family of proteins has been shown to regulate immune cell activity and thereby modulate the immune response-cell activation is regulated by a variety of immunomodulatory proteins. CD4^+^T-cell activation is mediated by the binding of peptide major histocompatibility complexes (pMHCs) on the surface of antigen-presenting cells (APCs) to the T-cell receptor (TCR). Following pMHC-TCR interaction and CD4^+^ T cell activation, co-stimulatory molecules such as CD28, inducible T cell co stimulator (ICOS) and CD40 ligand (CD40L) translocate to the surface of CD4^+^ T cells. Together, these co-stimulatory and co-inhibitory molecules regulate the duration and intensity of TCR-mediated signaling to promote an optimal immune response. Given the complexity of the immune response, precise regulation of the expression of co-stimulatory molecules, among other processes, is essential for a well-coordinated T cell response. Among other things, protein-mediated m6A modifications such as METTL3 regulate the expression of CD 40 L in human CD4^+^ lymphocytes and modulate CD4^+^ lymphocyte activation. However, CD40L is not the only co-stimulatory molecule expressed on activated CD4^+^ T cells, and co-inhibitory molecules contribute to the regulation of immune response. Studies of the mechanisms associated with m6A modifications in CD4^+^ lymphocytes can contribute to the clinical management of autoimmune diseases such as rheumatoid arthritis, systemic lupus erythematosus, and Scheuglen’s syndrome [84]. Regulatory T cells (Tregs) are heterogeneous subpopulations of helper T (Th) cells, which can be classified into thymic Tregs (tTreg) (originating from the thymus) as well as peripheral Tregs (pTreg) (originating from peripheral tissues) by constitutive expression of the transcription factor FoxP3, and play an important role in immune homeostasis by suppressing self-reactive effector T cells (Teff) [85, 86]. Therefore, therapies based on iTregs (regulatory T cells induced in vitro) have received increasing attention for their potential to treat autoimmune diseases and prevent transplant rejection. However, Tregs cells constitute only 10–15% of the CD4^+^T cell population; and iTregs are unable to maintain stable FoxP3 expression and rapidly lose their inhibitory activity, which are two major limitations for the clinical application of Tregs. Liu et al. demonstrated that METTL14 could regulate the stability and function of Tregs cells. The specific mechanism is that METTL14 defects in iTregs activate the mTOR-dependent pathway, increase p-mTOR and p-p70S6K, disrupt iTregs polarization, and downregulate FoxP3 expression. Meanwhile, inflammatory cytokines such as IFN-γ and IL-17a were up-regulated in cultured iTregs. METTL14 defects significantly impaired the inhibitory function of iTregs in vivo and in vitro, which revealed that METTL14 is a positive regulator of iTregs [87]. We review the mechanism of METTL protein family involvement in tumor immunity and summarize the role played by METTL protein family in immunotherapy in the following paper (Fig. 4). The relationship between METTL protein family and tumor immunity has received much attention and is a very promising research direction for the future.

### The role of METTL1 in tumor immunity

There is a significant correlation between METTL1 and regulatory T cell content in BRCA, KIRP, KIRC, LUAD, LIHC, PRAD and SARC. In TGCT, METTL1 was negatively correlated with resting mast cell, M2 macrophage, and M0 macrophage content, and showed significant positive correlations in activation memory CD4^+^T cells, CD8^+^T cells, follicular helper T cells, and naïve B cells. For multiple immunosuppressive agents, KDR and CD274 showed the most significant negative correlation with METTL1 in almost all cancers. Among MHC molecules and immunostimulators, most biomarkers were negatively correlated with METTL1, except for KIRP, LGG and TGCT. Three ICB therapeutic biomarkers (MSI, TMB, and CD274) showed significant correlation with METTL1 in a few cancers. METTL1 was negatively correlated with MSI and TMB in COAD but positively correlated with markers of HNSC, KICH, PRAD, and STAD, suggesting that METTL1 may have an impact on ICB therapeutic response in cancers such as HNSC. response. ICB treatment response was correlated with METTL1. METTL1 expression in the no/limited response group was significantly lower than that in the anti-PD-L1 cohort response group, suggesting that METTL1 expression may reflect the sensitivity to ICB therapy, but the difference was not significant. METTL1 may be effective in the treatment of HNSC and other cancers through the targeting of other immune checkpoints, including the T-cell Ig and ITIM structural domains (TIGIT), or the cytotoxic T lymphocyte-associated protein 4 (CTLA-4). protein 4 (CTLA-4)) to affect the response to ICB therapy, which requires further experimental evidence [88].

### The role of METTL3 in tumor immunity

METTL3 has been more studied in tumor immunity. JNK signaling promotes immune escape from BCa by regulating METTL3-mediated m6A modification of programmed death ligand 1 (PD-L1) [16]. In vitro and in vivo, activated JNK signaling enriches METTL3-mediated m6 A in the 3’-UTR of PD-L1 mRNA by binding c-Jun to the METTL3 promoter, allowing PD-L1 mRNA to be recognized by the m6A reader, IGF2BP1, to mediate RNA stability and PD-L1 expression levels that This in turn resists cytotoxicity of CD8^+^T cells and promotes tumor immune escape. METTL3 inhibition is a promising direction for targeting immunotherapeutic resistance in bladder cancer. Smoking is a major risk factor for bladder cancer because it activates JNK signaling. However, the effect of smoking on JNK signaling activation and METTL3 expression needs to be further investigated [89]. It has also been shown that METTL3 regulates the m6A modification of PD-L1, and knockdown of METTL3 in OSCC cells inhibits the activation of CD8^+^ T cells [90].

PD-L1 can bind to T cell PD-1, inhibit the immune recognition function and cytotoxicity of T cells against tumor cells, and promote immune evasion and tumor growth. Therefore, ICI therapies such as anti-programmed death receptor 1 (PD-1) therapy have dramatically changed the face of cancer treatment. However, many cancer patients have a low response rate to ICI therapy, for example, in ICI therapy for CRC, the objective remission rate of Regorafenib in combination with anti-PD-1 antibody for CRC was 33%. Clinical studies have shown that ICI treatment response is positively correlated with tumor-infiltrating T-cell (TIL) levels. However, many solid tumors lack sufficient T-cell infiltration, leading to immune evasion and further progression of the tumor, highlighting the need for effective recruitment and infiltration of tumor-targeted T-cells to the tumor site. The primary mechanism of T-cell infiltration is the response of G-protein-coupled receptors to chemokines, which facilitates the activation of integrins through an inside-out signaling pathway. Integrins can bind to ECM proteins to promote dynamic cytoskeleton assembly and the formation of filamentous pseudopods, invasive pseudopods, and pseudopod bodies, which allow T cells to move along collagen in the ECM with pseudopod amoeboid motility. Skin cancer extracellular acidification inhibits METTL3-mediated m6A modification and the level of its downstream target integrin β1 (ITGβ1), thereby suppressing T cell migration. This suggests that METTL3-m6A is an upstream epigenetic regulator in response to acidic TME and plays a key role in regulating T cell infiltration as well as T cell anticancer activity. Down-regulation of METTL3 activity or enhancement of ITGβ1 expression to increase T-cell infiltration within acidic TME are potential ways to improve the therapeutic efficacy of ICB [91]. However, another study showed that lactate accumulation in TME mediated K281 and K345 lactoylation in the zinc finger structural domain of METTL3 caused significant upregulation of METTL3 in CRC-infiltrating myeloid cells, which enhanced the immunosuppressive activity of tumor-infiltrating myeloid cells through the lactoylation-METTL3-m6A-JAK/STAT 3 axis and was associated with a poor prognosis in CRC patients. Therefore, the clinical application of METTL3 inhibitors is expected to have beneficial effects on CRC immunotherapy [92]. The above also confirms the complexity of METTL3 function in tumor ICI therapy.

**Fig. 5:**
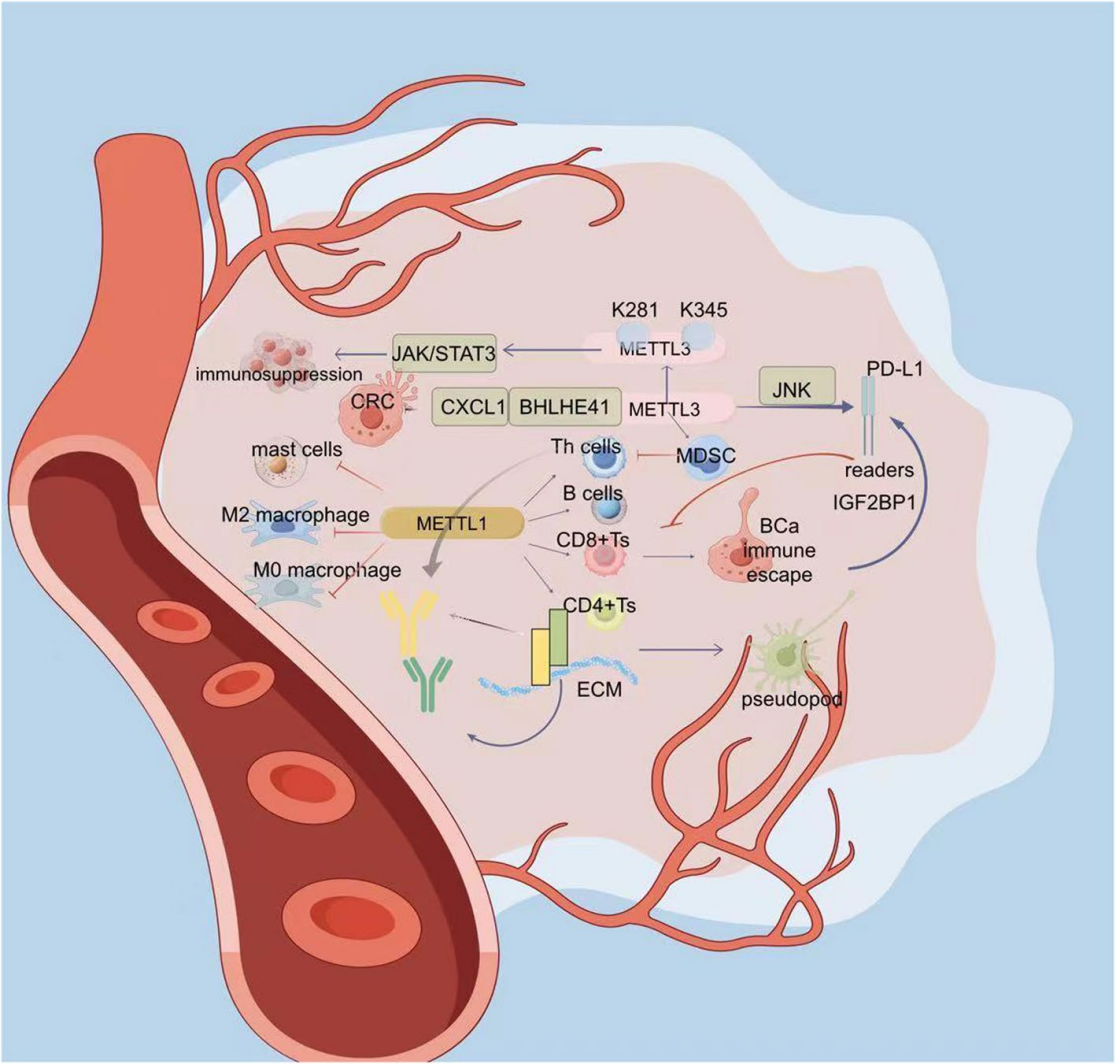
METTL3 significantly inhibited the proliferation of tumor cells in tumor microenvironment. In TGCT, METTL1 was negatively correlated with resting mast cell, M2 macrophage, and M0 macrophage content, and showed significant positive correlations in activation memory CD4^+^T cells, CD8^+^T cells, follicular helper T cells, and naive B cells. In vitro and in vivo, activated JNK signaling enriches METTL3-mediated m6 A in the 3’-UTR of PD-L1 mRNA, allowing PD-L1 mRNA to be recognized by the m6A reader, IGF2BP1, to mediate RNA stability and PD-L1 expression levels which in turn resists cytotoxicity of CD8^+^T cells and promotes tumor immune escape. Integrins can bind to ECM to allow T cells to move along collagen in the ECM with pseudopod amoeboid motility. Skin cancer extracellular acidification inhibits METTL3-mediated m6A modification and the level of its downstream target ITGβ1, thereby suppressing T cell migration. However, K281 and K345 lactoylation caused significant upregulation of METTL3 in CRC-infiltrating myeloid cells, which enhanced the immunosuppressive activity of tumor-infiltrating myeloid cells through the lactoylation-METTL3-m6A-JAK/STAT3 axis. METTL3 can promote colorectal carcinogenesis by inhibiting anti-tumor immunity through targeting the m6A-BHLHE41-CXCL1 axis. Down-regulation of METTL3 would reduce MDSC accumulation to maintain the activation and proliferation of CD4^+^T-cells and CD8^+^T-cells

Immunosuppression is a malignant tumor phenotype triggered by the accumulation of immunosuppressive cells. Immunosuppressive cells include myeloid-derived suppressor cells (MDSCs) and tumor-derived cytokines. MDSCs, of which there are two types, G-MDSCs and monocyte MDSCs, can strongly inhibit the antitumor activity of T cells and natural killer (NK) cells (especially G-MDSCs), impair antitumor immunity and trigger immune checkpoint inhibitor (ICI) resistance. Therefore, blocking MSDCs is a potential approach to improve the outcome of ICI therapy in patients with advanced CRC. METTL3 can promote colorectal carcinogenesis by inhibiting anti-tumor immunity through targeting the m6A-BHLHE41-CXCL1 axis. Down-regulation of METTL3 would reduce MDSC accumulation through the m6 A-BHLHE 41-CXCL1 /CXCR 2 axis to maintain the activation and proliferation of CD4^+^ T-cells and CD8^+^ T-cells, which play an inhibitory role in CRC. Meanwhile, depletion of MDSC by anti-Gr-1 antibody eliminated the tumor-promoting effect of METTL3 in vivo. The combination of METTL3 inhibition and anti-PD-1 is an effective direction for MSDC-blocking CRC therapy [93](Fig. 5).

### The role of METTL7A in tumor immunity

METTL7A plays an important role in tumor immunity. Multiple bioinformatics analyses have revealed the role played by METTL7A in tumor immunity from different perspectives. GSEA-GO analyses showed that METTL7A may influence tumor pathogenesis through leukocyte activation and adaptive immune response. GO and KEGG analyses revealed that differentially expressed genes in patients with high METTL7A expression and low METTL7A expression play an important role in antigen processing and presentation as well as adaptive immune response, suggesting that METTL7A may play a role in immune response and immune checkpoint inhibitor therapy. In immunotherapy, both tumor mutational load (TMB) and microsatellite instability (MSI) are important biomarkers of response to immunotherapy. In LUAD, METTL7A was negatively correlated with both TMB and MSI, suggesting that METTL7A is important in maintaining genomic stability. Meanwhile, METTL7A was negatively correlated with MSI in COAD and positively correlated with MSI in READ.METTL7A was positively correlated with immune scores in 12 cancers, and negatively correlated with immune scores in BLCA, CESC, LIHC, KIRC, and THCA.METTL7A is highly expressed in immune cells and is involved in immune cell infiltration. cell infiltration. Specifically, METTL7A showed increasing correlation with invasive immune cells such as B cells, CD4 + T cells and mast cells, and negative correlation with M0 macrophages, activated mast cells, activated memory CD4 + T cells, CD8 + T cells and follicular helper T cells in several tumors. In addition, METTL7A showed similar correlation with genes related to cancer immunotherapy [94]. METTL7B was also significantly associated with tumor immune cell infiltration [95].

### The role of METTL11B in tumor immunity

Cibersort analysis showed that METTL11B could affect immune cells such as Th17 cells, Th1 cells, Tgd cells, Tfhs, TEM, TCN, CD 56 bright NK cells, T helper cells, neutrophils, mast cells, NK cells, macrophages, IDCs, eosinophils, and DCs (all *P* < 0.001); Meanwhile, METTL11B also interacted with MSI-related molecules (PMS 1, MSH 6, PMS 2, MLH 1 and MLH 3). This suggests that METTL11B is closely associated with immune infiltration in CRC, but its specific mechanism needs to be proved by further experiments [76].

### The role of other members of the METTL protein family in tumor immunity

Inhibition of METTL14-mediated m6A mRNA modification improves the efficacy of anti-PD-1 therapy in CRC. Inhibition of METTL14 not only promotes the proliferation and accumulation of cytotoxic tumor-infiltrating CD8 + T cells, but also induces the secretion of IFNC, CXCL9, and CXCL 10, which enhances the efficacy of immunotherapy and inhibits cancer proliferation [96].

In CRC tissues and cell lines, decreased METTL16 expression and increased PD-L1 expression enhanced the process of CRC cell proliferation, migration and invasion. Overexpression of METTL16 reduces PD-L1 expression in CRC cells by inducing m6A modification and PD-L1 RNA degradation, which is an effective target for CRC immunotherapy [97].

METTL24 can participate in immune processes such as cytokine binding, NF-κB binding, MHC protein complexes and interleukin-12 action. Meanwhile, METTL24 is associated with some immune checkpoints, cytokines, chemokines and chemokine receptors. The level of immune cell infiltration in KIRC in 10 and the survival rate of patients with different immune infiltrations were also correlated with METTL24 [98].

## The role of METTL protein family as a biomarker in cancer diagnosis

### The role of METTL1 as a biomarker in cancer diagnosis

GSEA analysis showed that genes of proliferation- and metastasis-related pathways were more likely to be enriched in high METTL1 expression cohorts in KIRC, LGG, and LIHC, demonstrating that METTL1 is a poor patient prognosis biomarker in these cancers and can be used to assess patient survival [88]. tRNA N7 methylguanosine (m7G) methyltransferase complex components METTL1 and WD Repeat Domain 4 (WDR 4) expression levels were significantly elevated in human lung cancer samples and negatively correlated with patient prognosis [99].METTL1 and WDR 4 proteins were significantly up-regulated in HCC, which promotes HCC cell proliferation, migration, and invasion, and were negatively correlated with HCC patient survival at levels associated with a poor prognosis for HCC [100]. Under the regulation of PKB and ribosomal S6 kinase [101], METTL1 can catalyze 7-methylguanosine (m7G) modification in microRNAs and tRNAs, and exerts pro-HCC activity through the PTEN/AKT signaling pathway by inhibiting PTEN signaling. METTL1 induces the expression of NANOG and KLF4 [102], the latter Both were shown to be regulated by PTEN, and knockdown of NANOG downregulated the METTL1-mediated HCC malignant phenotype. However, the specific mechanism by which METTL1 regulates PTEN and whether METTL1 is involved in the stemness of HCC through PTEN-regulated NANOG requires further investigation [103]. upregulation of METTL1 has been associated with poor prognosis and HCC progression in HCC patients [102–104]. Through an unbiased proteomic screen of parental and lenvatinib-resistant HCC cells, two key tRNA m7 G methyltransferase complex components, METTL1 and WDR 4, were found to be significantly up-regulated in Lenvatinib-resistant HCC cells, with METTL1 being the catalytic enzyme and WDR 4 acting as a regulator. By testing HCC organoids for Lenvatinib sensitivity, high expression of METTL1 with WDR 4 proved to be more tolerant to Lenvatinib. Meanwhile, patients with low levels of METTL1 and WDR4 had a better prognosis for Lenvatinib treatment. Therefore, as a biomarker for predicting the efficacy of Lenvatinib, METTL1/WDR4 could be used for personalized treatment of HCC [105]. METTL1, which is highly expressed in HNSCC, promotes the development and malignancy of HNSCC by up-regulating the translation of overall mRNAs including the PI3K/AKT/mTOR signaling pathway, and has been found to alter the immune landscape, which is associated with the poor prognosis in HNSCC patients [106]. METTL1 is highly expressed in enamel cell tumors (AM) and significantly correlated with postoperative recurrence.METTL1 can promote invasive growth of AM through the mitogen-activated protein kinase (MAPK) signaling pathway, and is a poor prognostic factor for AM [107]. METTL1 is significantly up-regulated in esophageal squamous cell carcinoma, and its mediated tRNA m7 G modification promotes malignant progression of esophageal squamous cell carcinoma through the RPTOR/ULK 1/autophagy axis, and its high expression is associated with poor prognosis of patients [108]. mETTL1 expression is significantly higher in gliomas than in paracancerous tissues in both normal brain tissues and glioma tissues, and the expression of METTL1 rises with the grade of glioma. mETTL1 is a potential independent prognostic risk factor, with short survival and poor prognosis in patients with high expression [109].

### The role of METTL3 as a biomarker in cancer diagnosis

A meta-analysis showed that in various cancers, METTL3 carriers predicted worse OS compared to patients with lower METTL3 expression. METTL3 expression was higher in females than in males, and high METTL3 expression was associated with poorer differentiation [110]. Pan-cancer analysis showed that METTL3 expression was up-regulated in a variety of cancers, and higher METTL3 expression levels were significantly associated with poorer overall survival in ACC, KICH and LIHC, and poorer DFS in ACC, CESC, HNSC, KICH and LIHC. METTL3 has the highest potential as a biomarker in ACC, KICH and LIHC, but this needs further experimental confirmation [111].

Esophageal squamous cell carcinoma (ESCC) has a low early diagnosis rate, low 5-year survival rate of patients and poor prognosis [112, 113]. m6A was found to be up-regulated at the tissue and cellular levels in ESCC. mRNA and protein of METTL3 were found to be high in ESCC, and the level of mRNA expression was correlated with the degree of differentiation and gender of ESCC, and played a METTL3 content showed a positive correlation with the proliferation level of ESCC cells, which may promote ESCC progression by mediating m6 A modification via the p21 axis affecting the cell cycle. ROC curves showed that METTL3 had a high sensitivity and specificity for the diagnosis of ESCC (the area under the ROC curve was 0.8030, with a sensitivity of 75.00% and a specificity of 72.06%), and at the same time, METTL3 was closely associated with worse prognosis of tumor patients, proving that METTL3 is an important diagnostic marker for ESCC [114, 115]. Overexpression of METTL3 in lung cancer cells activates the PI3K/AKT/mTOR pathway and mTOR-mediated protein synthesis, promotes the proliferation and metastasis of cancer cells, and is a poor prognostic factor for lung cancer patients [116]. In the development of non-small cell lung cancer, both m6A and long chain non-coding RNAs (lncRNAs) play key regulatory roles. The specific mechanism is that METTL3 can m6A-modify the lncRNA ABHD11-AS1 to promote the proliferation of NSCLC cells with the Warburg effect [117]; METTL3 also increases the m6A level of RAC3 mRNA, which increases RAC3 mRNA stability and upregulates the cell migration of CAFs through the AKT/NF-κB pathway promotion [118], all of which are closely associated with poor prognosis of NSCLC patients.METTL3 was significantly upregulated in HCC and correlated with shorter overall survival of HCC patients [119]. Kaplan-Meier survival curve analysis showed that high METTL3 expression was associated with poor survival (including disease related survival (DSS)). survival (DSS), overall survival (OS), progression-free survival (PFS) and disease free interval (DFI), and recurrence-free survival (RFS) were poorly correlated, which is a prognostic independent predictor of hepatocellular carcinoma [120, 121]. METTL3-mediated methylation of Hsa_circ_0058493 m6A accelerates hepatocellular carcinoma progression and induces poorer prognosis through binding to YTHDC 1 [122]. Meanwhile, METTL3 upregulation in HCC marks Lenvatinib resistance and is a biomarker for predicting the efficacy of Lenvatinib in HCC [123]. The high expression of METTL3 in ICC mainly upregulates the m6A modification of NFAT5 mRNA, which recruits IGF2BP1 for the stabilization of NFAT5 mRNA. Elevated NFAT5 expression increases the expression of gluconeogenesis-related genes GLUT1 and PGK1, leading to enhanced aerobic glycolysis, proliferation and tumor metastasis in ICC.METTL3 is a poor prognostic factor for ICC patients [124]. In colorectal cancer (CRC), high expression of METTL3 promotes the proliferation, migration, and invasive process of CRC cells. Epitope transcriptomics microarrays revealed that the cell polarity regulator Crumbs 3 (CRB3) is a downstream target of METTL3. Knockdown of the METTL3 gene resulted in a significant decrease in CRB3 m6A levels, inhibition of CRB3 mRNA degradation, and increased CRB3 expression. METTL3 promotes colorectal cancer progression by regulating the m6 A-CRB3- Hippo axis, and CRC patients with high m6A or METTL3 levels have shorter overall survival. Meanwhile, METTL3 promotes CRC growth and metastasis through m6A modification of the coding sequence region of the YPEL5 transcript, which exerts epigenetic repression of YPEL5 [125]. Meanwhile, METTL3 plays an important role in promoting CRC angiogenesis through the METTL3/LINC 00662/VEGFA axis. The specific mechanism is that LINC 00662 and VEGFA RNAs have METTL3-rich sites. METTL3 can dually regulate the stability of LINC 00662 and VEGFA RNAs to maintain their expression and promote angiogenesis in CRC, which in turn facilitates CRC progression. The effect of METTL3 inhibitors on CRC angiogenesis has not yet been investigated, thus this is a promising direction [126]. METTL3 is considered a poor prognostic factor in CRC patients [127]. Interestingly, METTL3 exhibited its cancer suppressive effects in CRC by regulating the p38/ERK pathway, which suggests us that METTL3 plays a rather complex role in the regulation of colorectal cancer [128]. METTL3 is significantly upregulated in bladder cancer (BCa) and promotes Bca cell proliferation, migration, and invasion. METTL3 can be associated with RAS-related (RRAS) mRNA m6A sites and inhibit the transcriptional activity of RRAS. Meanwhile, YTHDF 2 recognizes the m6A site of RRAS and mediates the degradation of RRAS, promoting tumor growth and metastasis. Targeting the METTL3/RRAS/YTHDF 2 regulatory axis may prove to be a promising strategy for the diagnosis and treatment of BCa. However, the specific mechanisms underlying the rise in METTL3 levels in BCa and the correlation between METTL3 expression and patient prognosis remain to be explored [129]. Up-regulation of METTL3 expression in oral squamous cell carcinoma (OSCC) promotes OSCC cell proliferation, self-renewal, migration, and invasive processes, mainly through BMI1 m6 A methylation at the post-transcriptional level to facilitate the translation of BMI1 in OSCC. The METTL3-m6 A-BMI1 axis may serve as a prognostic biomarker or a therapeutic target for OSCC patients [130]. Meanwhile, METTL3 also regulates the m6A modification process of PRMT 5 and promotes PRMT 5 expression, which in turn promotes metastasis and proliferation of OSCC. However, the specific mechanisms and signaling pathways remain to be explored [90]. METTL3 is upregulated in prostate cancer (PCa) tissues and promotes MYC expression through m6A modification, which promotes PCa cell growth and progression [131]. METTL3 upregulation is more pronounced in Pca tissues with bone metastasis, which can increase the ITGB1 expression level to promote the recruitment of PCa cells to collagen I through m6A modification, thus enhancing cell motility and bone metastasis [132]. METTL3 is a poor prognostic factor for overall and disease-free survival in PCa.METTL3 can also enhance MYC (c-myc) expression to promote PCa cell proliferation, invasion, and migration by increasing the level of m6 A of MYC mRNA transcripts, which is a poor prognostic factor for overall and disease-free survival in patients with PCa [131]. Compared with paracancerous tissues, METTL3 expression was higher in cervical cancer tissues; METTL3 overexpression was closely associated with higher FIGO stage as well as poorer pelvic lymph node metastasis, 5-year recurrence-free survival, distant-metastasis-free survival, progression-free survival, and overall survival, and was an independent indicator of poor prognosis for patients with early-stage cervical cancer [133]. In vitro and in vivo, METTL3 overexpression significantly promotes the metastasis of cervical cancer cells. The specific mechanism was that METTL3 mediated the m6A modification of the untranslated region at the 5′ end of histone Tissue L (CTSL) mRNA, and in turn, the m6A reading protein Insulin-like Growth Factor 2 (IGF2BP2) binds to the m6A site, which enhances the stability of the CTSL mRNA, thus promoting the metastasis of cervical cancer cells. Highly expressed METTL3 is closely associated with poor prognosis in cervical cancer [134]. METTL3 is highly expressed in gastric cancer, promotes proliferation and migration of gastric cancer cells and correlates with poor prognosis of patients. METTL3 mediates the m6A modification of DEK mRNA, which binds to the DEK 3’UTR through m6A, resulting in an increase in the stability of DEK mRNA and promoting DEK expression, promoting gastric cancer cell growth and metastasis [135]. METTL3 is also an independent predictor of poor prognosis in esophageal and nasopharyngeal cancers [136–138], and is highly expressed and predictive of poor prognostic outcomes in cancers such as glioblastoma and uroepithelial carcinoma of the bladder [110, 139, 140].METTL3 is highly expressed and predictive of poor prognostic outcomes in Kidney renal clear cell carcinoma (KIRC) tissues, and METTL3 expression is significantly upregulated in Kidney renal clear cell carcinoma (KIRC) tissues. METTL3 regulates HHLA 2 expression mainly through m6A modification of HHLA 2 mRNA, and the METTL3/HHLA 2 axis promotes tumorigenesis in KIRC. Compared with patients with high METTL3 expression, patients with low METTL3 expression had better OS [141]. However, compared to paraneoplastic tissues, renal cell carcinoma (RCC) tissues had lower METTL3 expression, which inhibited cell proliferation, migration, and invasion. Moreover, METTL3 inhibits tumor growth by promoting cell cycle G0/G1 arrest. Higher expression of METTL3 may predict better survival outcomes in RCC patients [142]. METTL3 is usually considered to represent a diagnosed malignant phenotype with a poor prognosis, but the finding that METTL3 can act as a prognostic factor in RCC suggests that METTL3 has complexity in serving as a cancer biomarker.

### The role of METTL7A as a biomarker in cancer diagnosis

METTL7A expression is downregulated in lung adenocarcinoma (LUAD) and plays an important role in the diagnosis and prognosis of LUAD. In addition, METTL7A was significantly associated with the clinical phenotype of LUAD patients. In most cancers, METTL7A was associated with favorable Overall Survival. While in BRCA and LAML, METTL7A was identified as a high-risk factor. There was age variability as well as stage variability in METTL7A expression, with patients younger than 60 years of age exhibiting higher METTL7A expression in STAD, BRCA, and UCEC, while patients in the same cohort had lower METTL7A expression in LUAD than patients older than 60 years of age; METTL7A expression was higher in early stage (I/II) tumors than in advanced stage (III/IV) tumors. The above studies suggest that METTL7A is a potential early biomarker for LUAD diagnosis and follow-up [94]. METTL7A is closely related to methylation and lipid metabolism and can be involved in a variety of tumorigenesis and development. In renal clear cell carcinoma (KIRC), mesothelioma and sarcoma, low expression of METTL7 A was significantly associated with poor worst overall survival (OS), disease-specific survival (DSS) and progression-free interval (PFI). Among them, the potential of METTL7A as a diagnostic prognostic biomarker in KIRC is high, but the specific mechanism of METTL7A biomarker in cancer needs to be validated by further experiments [84].

### The role of METTL7B as a biomarker in cancer diagnosis

METTL7B is predominantly secreted intracellularly in lung adenocarcinoma (LUAD) tissues with significantly high expression, which can promote LUAD cell proliferation, migration, invasion, and cell cycle regulatory processes. High expression of METTL7B is closely associated with short survival of LUAD patients. According to the Kaplan-Meier plotter database, LUAD patients with low METTL7B expression have relatively good overall survival (OS) and disease-specific survival (DSS). Meanwhile, since METTL7B is also secreted in the extracellular region, it was demonstrated that METTL7B could be clinically applied as a serum diagnostic biomarker by comparing the METTL7B levels in clinical sera of tumor patients with those of healthy volunteers [95, 143]. mRNA and protein of METTL7B are both up-regulated in NSCLC and are involved in CCND1-associated cell cycle regulation. Elevated expression of METTL7B is associated with poor prognosis in NSCLC patients [144]. METTL7B is positively correlated with OS and DFS in patients with low-grade glioma (LGG) and immune cell infiltration of LGG; and participates in extracellular matrix (ECM) and immune-related pathways in LGG, contributing to cell proliferation, cancer progression and EMT. Therefore, METTL7B is a LGG prognostic marker [145].

### The role of METTL13 as a biomarker in cancer diagnosis

Tumor cells have a high rate of protein synthesis, and therefore dysregulation of translation mechanisms is common, making it a common cause of tumor-related diseases [146, 147]. Among them, translation elongation regulators such as METTL13 play an important role in the regulation of protein synthesis [148, 149]. METTL13 expression is higher in head and neck squamous carcinoma (HNSCC) than in paracancerous tissues, and in vitro and in vivo experiments have demonstrated that METTL13 enhances the translational efficiency of Snail, which plays an important role in EMT. The elevated level of METTL13 and the prognosis of cancer patients showed a negative correlation and was a prognostic marker for overall survival in HNSCC patients [126]. However, METTL13 is an oncogene of KIRC and can inhibit KIRC growth and metastasis. Knockdown and overexpression of METTL13 resulted in increased and decreased proliferation, viability, invasive migration ability, and epithelial-mesenchymal transition (EMT) of KIRC cells, respectively, and the specific mechanism was then that METTL13 down-regulated the PI 3 K/ AKT/mTOR/HIF-1α pathway and bound to c-Myc to inhibit c-Myc protein expression. Compared with paracancerous tissues, METTL13 is under-expressed in KIRC, and its low expression is closely associated with poor prognosis of KIRC, which is a potential diagnostic marker and therapeutic target for KIRC, and plays a role in the diagnosis of pathological features of KIRC and the prediction of patient prognosis [150].

### The role of METTL14 as a biomarker in cancer diagnosis

The expression level of METTL14 in renal cell carcinoma (RCC) is lower than that in normal tissues, and down-regulation of METTL14 expression predicts poor prognosis of renal cell carcinoma. METTL14 inhibits RCC cells by up-regulating the m6A level of NEAT1_1, which in turn down-regulates the expression of NEAT1_1 through the selective recognition of m6A markers on NEAT1_1 via YTHDF2 [151]. METTL14 was significantly downregulated in KIRC tissues. In vitro experiments verified that METTL14 inhibited the proliferation and migration of KIRC cells, while overexpression of METTL14 increased the enrichment of m6A on chromosome 10 Pten, increased Pten mRNA stability in a YTHDF 1-dependent manner, and acted as an anti-tumor effect, which is a good prognostic marker for KIRC [94]. The expression of METTL14 was decreased in acute myeloid leukemia (AML), which affects m6A modification and thus promotes AML development, proving that METTL14 is a prognostic biomarker for AML [152]. Downregulation of METTL14 expression correlates with advanced clinicopathological features and poor prognosis of oral squamous cell carcinoma (OSCC). The level of METTL14 expression is closely correlated with the T-stage and degree of differentiation of OSCC. In vivo and in vitro experiments demonstrated that METTL14 down-regulation promotes OSCC growth and cervical lymph node metastasis, and the specific mechanism is that METTL14 inhibits the autophagy-related gene RB 1CC 1 expression through m6 A-IGF 2BP 2-dependent manner, which exerts an inhibitory effect on OSCC. This suggests that METTL14 is closely related to the prognosis of OSCC patients and is a favorable survival factor for OSCC patients [153]. Both univariate and multifactorial Cox regression analyses showed that METTL14 was an independent favorable factor affecting the prognosis of colorectal cancer [154]. METTL14 expression was down-regulated in CRC, which could inhibit the malignant process of CRC and suppress the process of proliferation invasion and migration of CRC cells through SOX 4-mediated EMT process and PI3K/AKT signaling. stearoyl-CoA desaturase 1 (SCD1) is also a downstream target gene of METTL14-mediated m6 A modification, which can reduce the level of SCD1 mRNA through m6A modification of SCD1 mRNA in a YTHDF 2-dependent manner, and then inhibit the tumorigenic process of CRC through the SCD1-mediated Wnt/β-catenin signaling pathway [155]. Significant downregulation of METTL14 in gastric adenocarcinoma (STAD) predicted TNM stage in STAD as well as poorer overall survival. METTL14 inhibits PTEN expression through an m6 A modification-dependent mechanism, which in turn inhibits STAD growth and liver metastasis in vivo. IGF2BP2 and IGF2BP3 are potential binding proteins for PTEN m6A modification sites, prolonging STAD half-life of PTEN mRNA in cells, which was positively correlated with PTEN expression. Therefore, METTL14 is a potential biomarker for STAD prognostic and therapeutic targets, and METTL14 agonists have broad clinical applications in tumor therapy [156]. Meanwhile, METTL14 is down-regulated in cancers such as triple-negative breast cancer (TNBC) [157], esophageal cancer (EC) [158], osteosarcoma (OS) [16], hepatocellular carcinoma [121], and nephroblastoma [159], and its low expression has been associated with poor prognosis in cancer patients.

Most studies have shown that METTL14 can play a role in cancer inhibition, but some studies have also confirmed that METTL14 can promote tumorigenesis and development [160]. The expression of METTL14 is closely related to the survival of pancreatic cancer patients. The specific mechanism is that METTL14 overexpression triggers an increase in the level of p53 m6 A, while decreasing the mRNA and protein levels of PERP, which is associated with poor prognosis of patients.METTL14 is also a poor prognostic factor in breast cancer.METTL14 overexpression can promote tumor invasion and metastasis through the modification of hsa-miR 146 a-5 p by m6A [161]. METTL14 can participate in the LINC 00942-METTL14-CXCR 4/CYP 1B1 pathway to play a pro-BRCA role [162]. The AURKA gene can inhibit the degradation of METTL14 through the ubiquitination pathway, which also plays a pro-carcinogenic role [163]. The up-regulation of METTL14 plays a better diagnostic role in the peripheral blood screening of breast cancer [164]. Meanwhile, METTL14 and WTAP are upregulated in head and neck squamous cell carcinoma (HNSCC), which is instructive for prognostic prediction in HNSCC [95].

### The role of other members of the METTL family as a biomarker in cancer diagnosis

The N3-methylcytidine (m3C) gene, METTL2A, is highly expressed in breast invasive carcinoma (BRCA), and differential analysis showed that METTL2A was differentially expressed in cancer and paracancerous tissues more strongly than METTL6 and METTL8. The amplification rate of METTL2A was approximately 7-fold that of METTL6 [165]. Enrichment analysis showed that METTL2A mainly functions in DNA synthesis and cell proliferation pathways. The above analysis suggests that METTL2A is a poor prognostic factor for BRCA patients and is associated with poorer survival outcomes [166]. In HCC tissues and cells, METTL5 expression was increased. high METTL5 expression was associated with poor prognosis in HCC patients. Knockdown of METTL5 can inhibit PD-L1 expression and malignant cell behavior in HCC by suppressing the Myc pathway [167]. METTL6 was highly expressed in HCC tissues compared with adjacent non-tumor tissues, which was closely associated with poorer survival outcomes in HCC patients [168]. METTL11B expression was higher in CRC tissues than in paraneoplastic tissues, and in CRC patients, progression-free survival and overall survival were significantly higher in the low METTL11B-expressing group than in the high METTL11B-expressing group, respectively (both *P* < 0.05). METTL11B is closely associated with poor patient prognosis and is an independent risk factor affecting CRC prognosis [76]. METTL16 expression is up regulated in HCC, which promotes HCC cell proliferation, migration, and invasion, and its increased expression is associated with poor prognosis of HCC patients [169]. In BLCA patients, METTL16 was downregulated in bladder cancer (BLCA) compared to normal tissue adjacent to the cancer. Low expression of METTL16 was associated with higher TNM stage and poor prognosis in bladder cancer patients [170]. Compared to normal tissues, METTL24 expression was significantly lower in KIRC tissues, which correlated with lower survival in KIRC patients. This suggests that METTL24 is a protective factor in KIRC and can be used as a prognostic indicator for the diagnosis and treatment of KIRC [98]. Finally, we draw supplementary material 1 to summarize.

## The role of METTL protein family in cancer treatment

The role of the METTL family of proteins in a variety of cancers has led it to be viewed as a potential target for cancer therapy, playing a facilitating or inhibitory role in cancer chemotherapy, targeted therapy, immunotherapy, and other therapies (Table 2). Currently, drugs such as 5-fluorouracil (5-FU), cisplatin, and gemcitabine have become the standard of care in cancer treatment. However, accordingly, the resistance to these drugs also poses a considerable challenge to cancer treatment. Many studies have demonstrated that the METTL family of proteins plays an indispensable role in the resistance to these drugs. Interestingly, the METTL family of proteins can also regulate the therapeutic efficiency of certain drugs through mRNA epigenetic modifications. In addition, the role of METTL protein family inhibitors in cancer therapy is also emerging in basic clinical research, and the development and study of METTL protein family inhibitors is a new and promising direction. At the same time, existing research suggests that cancer-related healthcare beyond treatment is an issue that should not be ignored and that cancer healthcare accounts for a low proportion of total healthcare expenditure compared to the burden of the disease [171]. As personalized medicine becomes more prevalent in healthcare, the focus on healthcare is increasing [172, 173]. Thus, it is easy to see that cancer treatment and healthcare go hand in hand. As we further deepen our understanding of the mechanisms by which the METTL family of proteins regulates the development of cancer, this will contribute to the healthy development of treatment and healthcare. Currently, METTL1, METTL3, METTL7A, METTL7B, METTL14, and METTL16 have been reported in clinical studies, and the translational process of applying the basic research results of the METTL protein family to the clinic is crucial.

### The role of METTL protein family in therapy resistance

#### The role of METTL1 in therapy resistance

Tumor cell lines with higher METTL1 expression are more sensitive to drugs targeting chromatin histone methylation, ERK-MAPK, and Wnt signaling pathways, whereas relatively lower sensitivity to drugs targeting cell cycle, apoptosis, protein stability, and degradation signaling pathways is associated with resistance to these drugs. Thus, patients with higher METTL1 benefit from drugs targeting chromatin histone methylation, ERK-MAPK, and WNT pathways rather than cell cycle, apoptosis, and protein stability and degradation signaling pathways [88]. Lenvatinib, a tyrosine kinase inhibitor, is used as first-line therapy for advanced HCC. However, the development of resistance to Lenvatinib in HCC limits its efficacy and treatment duration. Lenvatinib-induced m7G tRNA modification enhances EGFR translation, which further triggers resistance [105]. In HeLa cells, knockdown of METTL1 significantly enhanced cell sensitivity to 5-FU chemotherapy [174].

#### The role of METTL3 in therapy resistance

METTL3 can regulate MALAT1 expression through m6A methylation modification of MALAT1, which in turn recruits E2F1 and activates downstream AGR2 expression to promote resistance to Adriamycin in breast cancer patients [175, 176]. METTL3 also accelerates the expression of pri-microRNA 221–3 through m6A-dependent manner to miR-221–3 p-mature conversion, which in turn negatively regulates the oncogene HIPK 2-mediated degradation of Che-1 and promotes drug resistance to Adriamycin in MCF-7 breast cancer cells [177]. Hormone receptor-positive/human epidermal growth factor receptor 2-negative (HR + HER2-) breast cancer chemotherapy is less efficient. METTL3 depletion was detected in HR + HER2- BC. METTL3 can regulate EMT through cyclin-dependent inhibitor kinase 1 A (CDKN 1 A); and METTL3 depletion can inhibit apoptosis by down-regulating Bax and thus caspase-3/-9/-8 signaling. reduce the efficiency of chemotherapy. Therefore, METTL3 is a potential therapeutic target for reversing chemoresistance in HR + HER2- BC and a good prognostic marker for HR + HER2- [178]. In gastric cancer, METTL3 promotes PARP1 mRNA stability and thus resistance to oxaliplatin in gastric cancer CD 133 + stem cells [179]. Chemoresistance to 5-fluorouracil (5-FU) is a major obstacle to the outcome of colorectal cancer (CRC) patients. Up-regulation of METTL3 expression was observed in 5-FU-resistant CRC cells, and inhibition of METTL3 inhibited glycolysis and restored chemosensitivity in 5-FU-resistant CRC cells. Specifically, METTL3 increased LDHA transcription by stabilizing hypoxia-inducible factor 1 α (HIF-1α) mRNA, and METTL3 also initiated the translation of LDHA mRNA through the recruitment of YTH structural domain family protein 1 (YTHDF 1), etc., and enhanced the expression of LDHA, which catalyzes the conversion of pyruvate to lactate, thereby triggering the glycolysis and 5-FU resistance of CRC cells. 5-FU resistance. Therefore, METTL3/LDHA-induced metabolic reprogramming is a potential therapeutic target for CRC resistance [180, 181]. Exosomes are extracellular vesicles that can regulate a variety of biological processes and can carry proteins, lipids, DNA and RNA. Among them, exosomes carrying miRNAs can be taken up by recipient cells, and exosomal miRNAs have been shown to affect tumor proliferation and metastasis. high expression of METTL3 in CRC promotes the binding of DGCR8 to pri-miR-181d-5p in an m6A-dependent manner, which results in increased expression of exosome-derived miR-181d-5p in cancer-associated fibroblasts (CAFs), and increased expression of miR-181d-5p in CAFs. expression is increased, and miR-181d-5p directly targets neurocalcin δ to inhibit the 5-FU sensitivity of CRC cells [182]. Li et al. used the Cancer Genome Atlas database to analyze and found that METTL3 expression was elevated in both primary and metastatic CRC lesions compared to normal tissues and that the expression of METTL3 was elevated in both CRC lesions when XALIX (OXA + Capecitabine) and FOLFOX (5-FU + OXA) were used as first-line regimens, patients with high METTL3 expression benefited less from chemotherapy [183]. METTL3-mediated upregulation of MALAT 1 through YTHDF 1/3 is associated with metastasis and drug resistance in lung cancer [184]. METTL3 is overexpressed in chemotherapy-resistant NSCLC tissues and is involved in NSCLC progression and chemoresistance by activating AKT1 protein through regulating the m6A level of AKT1 mRNA. Therefore, the METTL3-AKT1 axis is an effective therapeutic target for chemoresistance in NSCLC [185]. There is a strong association between betel nut and oral cancer. Betel nut chewers are much more likely to develop oral cancer than those who do not chew betel nut, and oral cancer patients who regularly consume betel nut have significantly lower five-year survival rates. As a result, the International Agency for Research on Cancer and the World Health Organization have classified betel nut/ betel nut as a Group 1 human carcinogen. Betel nut alkaloid betel nut, the most common alkaloid in betel nut extracts, is considered to be the major active carcinogen contributing to the pathogenesis and progression of oral cancer [186], which drives the pathologic progression of oral squamous cell carcinoma (OSCC). Arecoline-activated OSCC cell lines and oral squamous carcinoma tissues of betel nut chewers showed a significant up-regulation of METTL3, which induced a significant increase in OSCC through the METTL3/HIF-1α/MYC signaling pathway induced cisplatin resistance in OSCC [187]. Lenvatinib is a tyrosine kinase inhibitor (TKI), but its therapeutic efficacy is limited by drug resistance. In lenvatinib-resistant HCC cells, METTL3 undergoes significant up-regulation, which can regulate EGFR mRNA translation in an m6A-dependent manner, driving lenvatinib resistance in HCC through the METTL3-m6 A-EGFR axis [188]. m6A-mediated METTL3-ATP-mediated RHPN1-RAHAS1 modification accelerates cisplatin resistance in OC by activating the PI3K/AKT pathway to promote the proliferative invasive migratory process in ovarian cancer (OC) [189]. In addition, METTL3 may promote drug resistance in pancreatic cancer [110, 190].

#### The role of METTL7A in therapy resistance

Multiple myeloma (MM) is a malignant tumor in which plasma cells accumulate in the bone marrow [191], and the main therapeutic agents are the proteasome inhibitors Bortezomib or Carfilzomib, but MM is susceptible to resistance to these agents, which affects the therapeutic efficacy of treatment [192]. Bone marrow adipocytes have been shown to promote MM cell growth and lead to MM resistance by inhibiting chemotherapy-induced apoptosis of tumor cells [193]. The specific mechanism is that METTL7A mediates the m6A methylation of L0 C606724 and SNHG 1 isochronous non-coding RNA (LncRNA), which contributes to the interaction of LncRNA with RNA-binding proteins, which in turn promotes the enrichment of LncRNAs in adipocyte exosomes and mediates MM drug resistance. Meanwhile, MM cells can elevate the methylation level of METTL7A protein via EZH2, which promotes the packaging of LncRNA into adipocyte exosomes and further promotes MM resistance [194]. METTL7A also promotes MM methotrexate resistance through activation of the pro-survival signaling pathway and attenuation of reactive oxygen species accumulation in choriocarcinoma cells [84].

#### The role of METTL14 in therapy resistance

METTL14 shows low expression in CRC tissues and plays a role in CRC drug resistance. The specific mechanism is that downregulation of METTL14 leads to a decrease in m6A levels, inhibition of recognition and binding of pri-miR-17 by YTHDC2, which in turn promotes the stability of pri-miR-17 mRNA, increases the expression of pri-miR-17 and miR-17-5p, and inhibition of MFN 2 by overexpressed miR-17-5p, resulting in decreased mitochondrial fusion, decreased mitochondrial fission and enhanced mitochondrial autophagy, ultimately inducing reduced 5-FU sensitivity. The METTL14/miR-17- 5 p/MFN2 signaling axis plays a key role in inducing CRC 5-FU chemoresistance [195]. Oxidative stress induced by chemotherapeutic agents promotes m6A methylation modification of PHLDB2 by METTL14, upregulates PHLDB2 protein expression, and upregulated PHLDB 2 reduces the binding affinity between EGFR and the EGFR recycling protein Rab11A, stabilizes the epidermal growth factor receptor (EGFR), facilitates its nuclear translocation and inhibits inhibition of ubiquitin-mediated EGFR degradation, leading to activation of EGFR signaling and subsequent Cetuximab resistance in CRC [196].

Currently, cisplatin treatment is the standard of care for NSCLC, but cisplatin resistance is widely seen in NSCLC, greatly reducing treatment efficacy and patient survival [197]. METTL14 is highly expressed in NSCLC and is increased to a greater extent in cisplatin-resistant cells.METTL14 can be recognized by up-regulating the level of p53 m6A, recognizing DGCR8 and through DGCR8, it can be used for the expression of pri-miR-19 [198]. METTL14 can recognize DGCR8 by upregulating p53 m6A level, recognize DGCR8 and through DGCR8, recognize and process pri-miR-19a, promote the conversion of pri-miR-19a to miR-19a-5p and upregulate the latter, and target RBM24 with miR-19a-5p and downregulate its expression, thus reducing RBM 24 binding to AXIN 1 and inhibiting AXIN 1 transcription, and enhancing cisplatin resistance in NSCLC. Since AXIN1 is a core component of the Wnt pathway, overexpression can down-regulate the malignant phenotype of NSCLC and enhance NSCLC apoptosis [198], and silencing of AXIN1 induces NSCLC metastasis by accelerating the epithelial-mesenchymal transition process [199]. Therefore the effect of METTL14 on the Wnt pathway in NSCLC is a direction worth mining [200]. In pancreatic cancer, knockdown of METTL14 enhances the sensitivity of cancer cells to cisplatin and promotes apoptosis and autophagy by inhibiting AMPKα, ERK 1/2 and mTOR signaling pathways [201]. Similarly, inhibition of METTL14 also enhanced gemcitabine sensitivity of PC cells by downregulating CDA [202].

**Table 2:** The METTL protein family in tumor drug resistance

Member	Cancer type	Medicine	Effect	Reference
METTL1	Pan cancer	Drugs targeting cell cycle, apoptosis, protein stability and degradation signaling pathways	Reduces drug sensitivity, induces drug resistance	[88]
	Hepatocellular carcinoma	Lenvatinib	Enhances EGFR translation, triggers drug resistance	[105]
	Cervical cancer	5-FU	Decreases chemosensitivity	[174]
METTL3	Breast cancer	Adriamycin	Regulates MALAT1 expression, recruits E2F1 and activates downstream AGR2 expression	[175]
			Accelerates the conversion of pri-microRNA 221–3 to miR-221–3 p maturation in an m6A-dependent manner and negatively regulates oncogene HIPK 2-mediated degradation of Che-1	[177]
	Gastric cancer	Oxaliplatin	Promotes PARP1 mRNA stability and thus promotes the resistance to oxaliplatin in gastric cancer CD 133 + stem cells	[179]
	Colorectal cancer	5-FU	Stabilizes HIF-1α mRNA by m6A modification to increase LDHA transcription; recruits YTHDF 1, which initiates translation of LDHA mRNA and promotes LDHA-catalyzed conversion of pyruvate to lactate, triggering CRC glycolysis and 5-FU resistance.	[180]
			Promotes DGCR8 binding to pri-miR-181d-5p in an m6A-dependent manner, results in an expansion of miR-181d-5p expression, and miR-181d-5p directly targets neurocalcin δ to inhibit 5-FU sensitivity in CRC cells	[182]
		XALIX（OXA + Capecitabine）and FOLFOX (5-FU + OXA)	Patients have less benefit from chemotherapy	[183]
	Oral squamous cell carcinoma	Cisplatin	Induces cisplatin resistance in OSSCC via the METTL3/HIF-1α/MYC signaling pathway	[187]
	Hepatocellular carcinoma	Lenvatinib	Regulates EGFR mRNA translation in an m6A-dependent manner and drives lenvatinib resistance in HCC through the METTL3-m6 A-EGFR axis	[188]
	Ovarian cancer	Cisplatin	Modifies RHPN1-RAHAS1 then activates the PI3K/AKT pathway	[189]
METTL7A	Multiple myeloma	Bortezomib/Carfilzomib	Mediates m6A methylation of LncRNA and promotes LncRNA enrichment in adipocyte exosomes	[194]
			Elevates METTL7A protein methylation levels via EZH2 promote LncRNA packaging into adipocyte exosomes	
	Pan cancer	Methotrexate	Activates pro-survival signaling pathways and attenuates reactive oxygen species accumulation in choriocarcinoma cells	[84]
METTL14	Colorectal cancer	5-FU	Down-regulation of METTL14 leads to a decrease in m6A levels, inhibits the recognition and binding of pri-miR-17 by YTHDC 2, which in turn promotes the stabilization of pri-miR-17 mRNA, increases the expression of pri-miR-17 and miR-17-5p, and overexpression of miR-17-5p inhibits MFN 2, leading to a decrease in mitochondrial fusion, an enhancement of mitochondrial fragmentation and mitochondrial autophagy, and ultimately induces a decrease in 5-FU sensitivity	[195]
		Cetuximab	Oxidative stress promotes m6A methylation modification of PHLDB2 by METTL14, upregulates PHLDB2 protein expression, reduces the binding affinity between EGFR and Rab11A, stabilizes EGFR, promotes its nuclear translocation and inhibits inhibition of ubiquitin-mediated EGFR degradation, and activates EGFR signaling and cetuximab resistance	[196]
	Non-small cell lung cancers	Cisplatin	Up-regulates the m6A level of p53, recognizes DGCR8 and recognizes and processes pri-miR-19a via DGCR8, promotes the conversion of pri-miR-19a to miR-19a-5p and up-regulates the latter, down-regulates RBM24, reduces the binding of RBM24 to AXIN 1, inhibits AXIN 1 transcription, and enhances NSCLC cisplatin resistance	[197]

### Role of METTL protein family in modulation of therapy efficiency

The METTL protein family plays an important role in regulating the efficiency of cancer therapy by modulating mRNA and tRNA post-transcriptional modification processes. Differential expression of METTL1 has been shown to modulate the therapeutic efficacy of antitumor drugs. METTL1 overexpression enhances the cytotoxicity of cisplatin on cisplatin-resistant CRC cells via mir-149-3p action via the S100 A4/p53 signaling pathway to increase cytotoxic effects [203]. It can also increase the sensitivity of antitumor drugs such as cisplatin and 5-fluorouracil in Hela cells, CRC cells [174, 203]. METTL1 expression is lower in cisplatin-resistant (CR-CC) cells compared to cisplatin-sensitive CC (CS-CC) cells, and overexpression of METTL1 increases chemosensitivity to cisplatin in CRC cells by regulating the miR149-3p/S100A4/p53 axis. However, the specific mechanism of METTL1’s action with miR-149-3 p remains to be further explored [126]. Drug sensitivity analysis showed that Trifluridine, PD 407,824 and Taselisib are effective drugs for the treatment of METTL2A-positive BRCA patients [166]. METTL3 can increase the sensitivity of gastric cancer to mTOR inhibitors by promoting the maturation of the miR-17-92 cluster [204]. Allocryptopine is an isoquinoline alkaloid derived from Macleaya cordata that can be used in oral squamous cell carcinoma (OSCC) therapy. Allocryptopine down-regulates METTL3 expression in a dose-dependent manner and inhibits the m6A modification process of PTCH1, which in turn inhibits the proliferation and epithelial-mesenchymal transition of OSCC cells through the m6A modification-mediated Hedgehog signaling pathway, and attenuates the oncogenic behavior of OSCC [205]. Encapsulation of Cromolyn in chitosan nanoparticles (CSNPs) could lead to further enhancement of the former’s therapeutic effect and bioavailability. Cromolyn CSNP can significantly downregulate METTL3 expression and inhibit the m6A process in MCF-7 cells, which suggests that Cromolyn CSNP is a novel epigenetic anticancer drug [206]. p53 inactivation directly promotes the development of HCC and chemotherapy resistance, so p53 reactivation through epigenetic modification is a promising HCC therapeutic research direction. Studies have shown that the combination of vascular endothelial growth factor receptor inhibitors and p53 activators can exert synergistic anti-cancer effects. The combination of RG7112, a p53 activator, and Apatinib, a vascular endothelial growth factor receptor inhibitor, significantly reduced the binding of METTL3 to p53 mRNA, down-regulated the methylation of p53 m6A, activated p53, and significantly increased the ratio of Bax/Bcl-2 to induce apoptosis in HCC cells, thus exerting anti-HCC effects. However, the mechanism of interaction between METTL3 and p53 has not been fully elucidated, and the METTL3-m6A-p53 axis is a potential target for HCC treatment, and the co-administration of RG7112 and Apatinib is an HCC therapy that takes into account the safety and efficacy of HCC [119]. Metformin attenuates multiple myeloma (MM) cell proliferation and promotes apoptosis by inhibiting METTL3-mediated m6A modification of THRAP3, RBM25, and USP4, as well as their protein expression levels [207]. METTL3 promotes homologous recombinational repair and modulates the response to chemotherapy in breast cancer by upregulating the EGF/RAD51 axis through m6A modification. Knockdown of METTL3 increases the sensitivity of BC cells to adriamycin, promotes the accumulation of DNA damage in BC cells, and induces BC cell death [123]. Carbon-ion radiotherapy is a radical non-surgical treatment with a high rate of localized control and no serious adverse effects, which mainly triggers difficult-to-repair DNA double-strand breaks, inducing cell death and inactivation such as apoptosis, necrosis, autophagy, premature senescence, accelerated differentiation, delayed reproductive death of progeny cells and bystander cell death. In NSCLC, after Carbon-ion radiotherapy, METTL3 and its mediated m6A modification levels were up-regulated, inhibiting the degradation and enhancing the expression of H2AX mRNA, promoting DNA damage repair, and thus promoting the process of proliferation, invasion, and migration of NSCLC cells and counteracting Carbon-ion radiotherapy [208]. Drug sensitivity analysis showed that METTL7A was negatively correlated with LRRK2 inhibitors, histone deacetylase inhibitors, PI4KIII β inhibitors, and BET inhibitors, suggesting that METTL7A may be a good predictor of drug efficacy. However, its specific mechanism still needs to be further investigated [94]. The antitumor effect of METTL13 on ccRCC can be used in renal cancer treatment. On the one hand, it is feasible to use METTL13 agonists. On the other hand, the application of the inhibitory effect of METTL13 on HIF-1α, c-Myc, and metabolism-related genes provides a new strategy for ccRCC treatment [150]. METTL16 co-localized with the prostate transmembrane protein androgen induced-1 (PMEPA 1) with the BLCA cells by binding to the m6 A site of the 3 ‘-UTR of PMEPA1, decreasing its mRNA stability as well as protein expression, thereby increasing cisplatin sensitivity and inhibiting the proliferation of bladder cancer cells through the PMEPA 1-mediated autophagy pathway. And under hypoxic conditions hypoxia-inducible factor 2α (HIF-2α) exerts a pro-tumorigenic effect by binding to the METTL16 promoter region and inhibiting its transcription. Therefore, METTL16 and its upstream and downstream play an important role in improving the therapeutic efficiency of BLCA [170].

### The inhibitors of the METTL protein family

The METTL family of proteins has received much attention for its complex regulatory network in cancer development and metastasis. It is up- and down-regulated in a wide range of cancers and exerts pro- or anti-tumorigenic effects. Cancer therapeutic regimens such as targeted therapies and immunotherapies have been developed for the METTL family, which have been shown to play a positive role in the treatment of a variety of cancers. Currently, the development of METTL protein family inhibitors has received extensive attention. However, the development of METTL inhibitors has been mainly focused on METTL3, and therefore not enough research has been conducted on other members of the family. At the same time, the research on METTL inhibitors mainly focuses on the synthesis and screening of the inhibitors, while the exploration of the specific mechanism by which the inhibitors target the METTL family to participate in cellular signaling is not much, which is also a direction that future researchers need to work on (Supplementary material 2).

Early reported METTL3 inhibitors are SAM analogs, such as cmpd2, sinefungin, and cpd-564 [132, 209]. The mechanism of inhibition of SAM analogs-associated METTL inhibitors is mainly through competitive inhibition by structural overlap of METTL3 in complex with the inhibitor, SAM and SAH. However, SAM analogs have the disadvantages of poor cell permeability and low selectivity for other methyltransferases. Therefore, researchers have worked on finding and synthesizing non-SAM analogs of METTL3 inhibitors [210].

STM2457, a potent non-SAM analog METTL3 inhibitor, is also the first RNA methyltransferase modulator to exhibit antitumor activity and therapeutic efficacy in vivo. STM2457 plays a role in the treatment of myelogenous leukemia by specifically targeting a key stem cell subset of AML, and by pharmacological inhibition of METTL3 resulted in AML mouse models of impaired implantation and prolonged survival in AML mouse models [4]; and also synergistically inhibits METTL3 activity with gemcitabine, all of which suggest that reprogramming RNA epigenetic modifications may be a potential therapeutic strategy [124, 211]. Moreover, STM2457 is expected to be used in the treatment of NSCLC. STM2457 upregulates PD-L1 both in vivo and in vitro, which may improve the immunotherapy outcome based on PD-L1 upregulation in NSCLC treatment. Combination of STM2457 and anti-PD-1/PD-L1 treatment to improve the immunotherapy benefit is very feasible [212]. In vivo experiments demonstrated that in HCC STM2457 could overcome Lenvatinib resistance by inhibiting EGFR translation in Lenvatinib-resistant HCC (HCC-LR) cells, suggesting that METTL3 is a potential therapeutic target for overcoming Lenvatinib resistance in HCC. The combination of METTL3 inhibitor STM 2457 with Lenvatinib is promising in HCC treatment [188]. In addition, STC-15, a derivative of STM2457, is the first clinical candidate for human oral formulation targeting METTL3 and has entered a phase I clinical trial (NCT 05584111).

Non-SAM analogs such as UZH 2 and UZH 1a have also been reported as METTL3 inhibitors in previous studies. Among them, UZH 1a mainly exerts its inhibitory effect through strong van der Waals forces and the formation of hydrogen bonds with the polar group of METTL3. UZH 2 is a further optimized form of UZH 1a, which also exerts its inhibitory effect through hydrogen bonding.However, none of them have been clinically studied, and their adverse effects and drug safety are unknown [213, 214]. natural products such as quercetin also inhibit METTL3. Quercetin, a flavonol-type compound, is one of the most abundant of our food one of the natural polyphenols, which exerts inhibitory effects by filling SAM pockets. It is the first METTL3 inhibitor derived from a natural product and the most potent METTL3 inhibitor, inhibiting METTL3 activity and thus reducing m6A levels without affecting m5 C/C levels, and inhibiting the proliferation of four different tumor cell lines at micromolar IC_50_ levels. However, quercetin requires further studies to demonstrate its METTL3 specificity and whether it influences the expression levels of other RNA modifications. In addition, the potential use and in vivo efficacy of quercetin as a METTL3 inhibitor need further investigation. Structural modification and optimization of quercetin is a direction for future METTL3 inhibitor development [215]. Mutagenic inhibitors such as 4-[2-[5-chloro-1-(diphenylmethyl)-2-methyl-1 H-indol-3-yl]-ethoxy] benzoic acid (CDIBA) and eltrombopag reversibly and non-competitively interact with the METTL3/METTL14 complex and can be used as METTL3 inhibitors. competitively interact with the METTL3/METTL14 complex, which can be used as an alternative approach to inhibit METTL3 activity. In addition, Manna et al. identified hesperidin as a potent inhibitor of METTL3 by computerized screening and molecular dynamics simulations [183].

## Conclusions and future directions

In summary, METTL proteins are a family of proteins named by their structural features, which can participate in nucleic acid and protein methylation modification, are abnormally expressed in a variety of tumors, and are associated with biological behaviors such as tumor proliferation, cell cycle, invasive migration, apoptosis and autophagy. The effects of different METTL proteins on tumors are characterized by the duality of promotion and inhibition, which may be determined by the different expression or the state of the tumor microenvironment, and the specific mechanisms need to be studied. Therefore, in-depth analysis of the role of METTL proteins in tumor evolution and related mechanisms is an important issue to be resolved in this field, and more basic and clinical studies are needed in the future to further elucidate the specific mechanisms of METTL proteins, which is expected to become a marker for the diagnosis of tumors and prognosis, and may also become a new target for the treatment of cancer pain.

With the increasing research on this family, the emergence of drug-targeted therapies against METTL proteins has provided new opportunities for targeted tumor therapy. However, on the one hand, as METTL family proteins, as important components of multiprotein complexes, are susceptible to multiple acting components and play different roles under different conditions. On the other hand, the structural similarity of family members becomes a challenge for targeted therapy. All these factors will affect its application as molecular markers and drug action targets. In the future, it is necessary to comprehensively and systematically study the role of the METTL protein family in cancer and related signalling pathways, to construct interaction networks and to reveal its specific mechanism of action in cancer, so as to provide new ideas for targeted therapy of cancer. In addition, there is a need to further search for natural bioactive compounds that can replace small molecule inhibitors to reduce the toxic side effects on normal cells. Meanwhile, RNAs targeting METTL family proteins will be used as a new means of targeting cancer treatment in combination with radiotherapy, chemotherapy and other drugs.

Although current research has only revealed a small part of the role of the METTL protein family in tumorigenesis and progression, more and more data are gradually unravelling the mysteries of the METTL proteins. As we gain a more comprehensive understanding of the function of METTL proteins, it will provide new diagnostic and therapeutic strategies for targeted interventions in the future.

## Supplementary Information

Supplementary Material 1.

Supplementary Material 2.

## Data Availability

No datasets were generated or analysed during the current study.

## Abbreviations

METTL: Methyltransferase-like
RP: Retinitis pigmentosa
ID: Intellectual disability
SAM: S-adenosylmethionine
MTD: Methyltransferase domain
TME: Tumor microenvironment
pMHCs: peptide major histocompatibility complexes
APCs: antigen-presenting cells
TCR: T-cell receptor
ICOS: inducible T cell co stimulator
CD40L: CD40 ligand
Tregs: Regulatory T cells
Th: helper T cells
tTregs: thymic Tregs
pTreg: peripheral Tregs
Teff: effector T cells
PD-L1: Programmed death ligand 1
PD-1: programmed death receptor 1
TIL: tumor-infiltrating T-cell
ITGβ1: integrin β1
MDSCs: myeloid-derived suppressor cells
CRC: Colorectal carcinoma
TMB: Tumor mutation burden
WDR 4: WD Repeat Domain 4
HCC: hepatocellular carcinoma
m^7^G: 7-methylguanosine
AM: Ameloblastic fibroma
ESCC: Esophageal squamous cell carcinoma
lncRNAs: long chain non-coding RNAs
DSS: disease related survival
OS: overall survival
PFS: progression-free survival
DFI: disease free interval
RFS: recurrence-free survival
CRB3: Crumbs 3
BCa: bladder cancer
OSCC: oral squamous cell carcinoma
PCa: prostate cancer
CTSL: Cathepsin L
IGF2BP2: Insulin-like Growth Factor 2
KIRC: Kidney renal clear cell carcinoma
RCC: renal cell carcinoma
LUAD: lung adenocarcinoma
PFI: progression-free interval
LGG: low-grade glioma
HNSCC: head and neck squamous carcinoma
EMT: epithelial-mesenchymal transition
AML: acute myeloid leukemia
STAD: gastric adenocarcinoma
TNBC: triple-negative breast cancer
EC: esophageal cancer
m3C: N3-methylcytidine
BRCA: breast invasive carcinoma
5-FU: 5-fluorouracil
HIF-1α: hypoxia-inducible factor 1α
HIF-2α: hypoxia-inducible factor 2α
YTHDF 1: YTH structural domain family protein 1
CAFs: cancer-associated fibroblasts
TKI: tyrosine kinase inhibitor
OC: ovarian cancer
MM: Multiple myeloma
EGFR: epidermal growth factor receptor
CSNPs: chitosan nanoparticles
TEK: Tyrosine kinase endothelial
