# Supplementary material for: METTL protein family: focusing on the occurrence, progression and treatment of cancer
Source: Biomark Res. 2024 Sep 17;12:105. doi: 10.1186/s40364-024-00652-3 (PMC11409517; doi:10.1186/s40364-024-00652-3)
Supplement: Supplementary file 1 — Supplementary Material 1. [file 40364_2024_652_MOESM1_ESM.docx]

**Supplementary material 1:** The role of METTL protein family as a biomarker in cancer

| Member | Cancer type | METTL protein expression | Detection method | Reference |
| --- | --- | --- | --- | --- |
| METTL1 | Lung cancer | High expression | WB, IHC, TCGA analysis | ^99^ |
|  | Hepatocellular carcinoma | High expression | RNA‐seq, polyribosome‐associated mRNA‐qPCR, TCGA analysis, IHC, Northern blot, Western blot | ^100-105^ |
|  | Head and neck squamous cell carcinoma | High expression | TCGA analysis, Survival analysis, IHC, Northern blot, Western blot | ^106^ |
|  | Ameloblastoma | High expression | Survival analysis, IHC, Western blot, [Northern blot](https://www.sciencedirect.com/topics/neuroscience/northern-blot), Northwestern blot, qRT-PCR | ^107^ |
|  | Esophageal squamous cell carcinoma | High expression | IHC, WB | ^108^ |
|  | Glioma | High expression | Pan-cancer analysis, Correlation analysis, RT-PCR, IHC, RNA-seq, WB | ^109^ |
| METTL2A | Breast invasive carcinoma | High expression | Pan-Cancer Analysis, Analysis of Variance, CPTAC Database Mining, IHC, Univariate Analysis, Multivariate Analysis, Clinical Sample Analysis | ^165, 166^ |
| METTL3 | Esophageal squamous cell carcinoma | High expression | IHC, WB | ^112-115^ |
|  | Lung cancer | High expression | MeRIP-Seq, WB | ^116-118^ |
|  | Hepatocellular carcinoma | High expression | Pan-cancer analysis, Survival analysis | ^119-123^ |
|  | Intrahepatic cholangiocarcinoma | High expression | RT-PCR, IHC, WB | ^124^ |
|  | Colorectal cancer | High expression | Differential analysis, WB, IHC, Immunofluorescence | ^125-128^ |
|  | Bladder cancer | High expression | RT-qPCR, WB | ^129^ |
|  | Oral squamous cell carcinoma | High expression | Analysis of variance, Survival analysis, IHC | ^90, 130^ |
|  | Prostatic cancer | High expression | qRT-PCR, WB | ^131, 132^ |
|  | Cervical cancer | High expression | qRT-PCR, WB, IHC | ^133, 134^ |
|  | Gastric cancer | High expression | Analysis of variance, IHC, WB | ^135^ |
|  | Kidney renal clear cell carcinoma | High expression | Analysis of variance, IHC, WB | ^141^ |
|  | Renal cell carcinoma | Low expression | Analysis of variance | ^142^ |
| METTL5 | Hepatocellular carcinoma | High expression | Analysis of variance, Enrichment analysis, qRT-PCR, WB | ^167^ |
| METTL6 | Hepatocellular carcinoma | High expression | Analysis of variance, Enrichment analysis, WB | ^168^ |
| METTL7A | Lung adenocarcinoma | Low expression | Pan-cancer analysis, Survival analysis, IHC | ^94^ |
|  | Kidney renal clear cell carcinoma | Low expression | PPI network, GO enrichment analysis, Analysis of variance, Survival analysis | ^84^ |
| METTL7B | Lung adenocarcinoma | High expression | Analysis of variance, Survival analysis, IHC, WB | ^95, 143^ |
|  | Non-small cell lung cancers | High expression | Analysis of variance, Survival analysis, IHC, WB | ^144, 145^ |
| METTL11B | Colorectal cancer | High expression | TCGA database mining, Univariate analysis | ^76^ |
| METTL13 | Head and neck squamous cell carcinoma | High expression | Analysis of variance, Survival analysis, IHC, WB | ^126^ |
|  | Kidney renal clear cell carcinoma | Low expression | Analysis of variance, qRT-PCR, IHC, WB | ^150^ |
| METTL14 | Renal cell carcinoma | Low expression | Analysis of variance, Survival analysis, IHC, WB | ^151^ |
|  | Kidney renal clear cell carcinoma | Low expression | MRI | ^94^ |
|  | Acute myeloid leukemia | Low expression | Case-control study | ^152^ |
|  | Oral squamous cell carcinoma | Low expression | Analysis of variance, Survival analysis, IHC, WB, qRT-PCR | ^153^ |
|  | Colorectal cancer | Low expression | Analysis of variance, Survival analysis, IHC, WB, qRT-PCR | ^154, 155^ |
|  | Stomach adenocarcinoma | Low expression | Analysis of variance, Survival analysis, IHC, WB | ^156^ |
|  | Triple-negative breast cancer | Low expression | Analysis of variance, Survival analysis | ^157^ |
|  | Esophageal carcinoma | Low expression | Bioinformatics analysis | ^158^ |
|  | Osteosarcoma | Low expression | Analysis of variance, IHC | ^16^ |
|  | Hepatocellular carcinoma | Low expression | Pan-cancer analysis, Survival analysis | ^121^ |
|  | Nephroblastoma | Low expression | Case-control study | ^159^ |
|  | Pancreatic cancer | High expression | Analysis of variance, Survival analysis, IHC, WB | ^161^ |
|  | Breast Cancer | High expression | qRT-PCR | ^162-164^ |
|  | Head and neck squamous cell carcinoma | High expression | Analysis of variance, qRT-PCR | ^95^ |
| METTL16 | Hepatocellular carcinoma | High expression | Analysis of variance, Survival analysis, qRT-PCR | ^169^ |
|  | Bladder cancer | Low expression | Analysis of variance, Survival analysis, IHC, WB | ^170^ |
| METTL24 | Kidney renal clear cell carcinoma | Low expression | Analysis of variance, Survival analysis, IHC | ^98^ |
