# Supplementary material for: METTL protein family: focusing on the occurrence, progression and treatment of cancer
Source: Biomark Res. 2024 Sep 17;12:105. doi: 10.1186/s40364-024-00652-3 (PMC11409517; doi:10.1186/s40364-024-00652-3)
Supplement: Supplementary file 2 — Supplementary Material 2. [file 40364_2024_652_MOESM2_ESM.docx]

**Supplementary material 2.** The inhibitors of METTL protein family (METTL3)

| Inhibitor | Inhibitor type | IUPAC Name | Structures | References |
| --- | --- | --- | --- | --- |
| Cmpd2 | SAM analogs | 5-bromo-N-[(2S,4R)-2-[(4-chlorophenyl)methyl]piperidin-4-yl]-1H-indole-3-carboxamide | 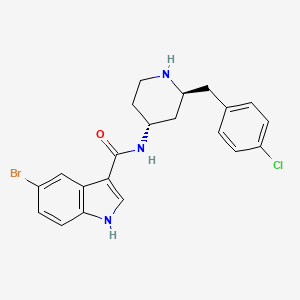 | ^132, 209^ |
| Sinefungin | SAM analogs | (2S,5S)-2,5-diamino-6-[(2R,3S,4R,5R)-5-(6-aminopurin-9-yl)-3,4-dihydroxyoxolan-2-yl]hexanoic acid | 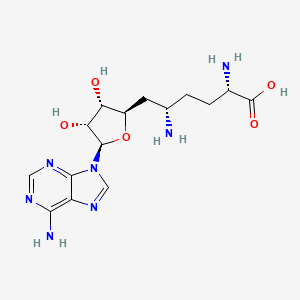 | ^132, 209^ |
| Cpd-564 | SAM analogs | (2S)-2-amino-4-[[(2S,3S,4R,5R)-3,4,5-trihydroxyoxolan-2-yl]methylsulfanyl]butanoic acid | 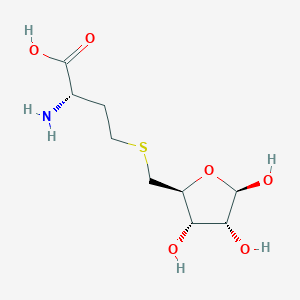 | ^132, 209^ |
| STM2457 | Non-SAM analogs | N-[[6-[(cyclohexylmethylamino)methyl]imidazo[1,2-a]pyridin-2-yl]methyl]-4-oxopyrido[1,2-a]pyrimidine-2-carboxamide | 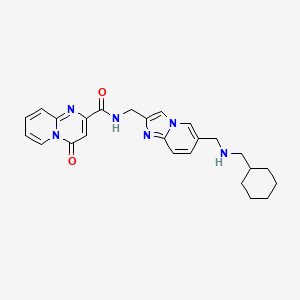 | ^124, 188, 211, 212^ |
| STC-15 | Non-SAM analogs | -[[2-[(cyclobutylmethylamino)methyl]-1H-indol-6-yl]methyl]-4-oxopyrido[1,2-a]pyrimidine-2-carboxamide | 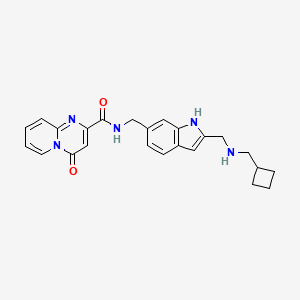 | ^188^ |
| UZH 2 | Non-SAM analogs | 4-[4-[(4,4-dimethylpiperidin-1-yl)methyl]-2,5-difluorophenyl]-9-[6-(methylamino)pyrimidin-4-yl]-1,4,9-triazaspiro[5.5]undecan-2-one | 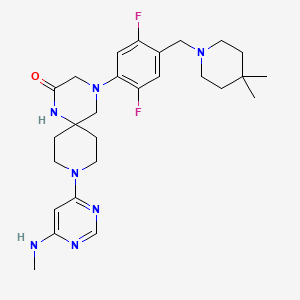 | ^213, 214^ |
| UZH 1a | Non-SAM analogs | N-[[(3R)-1-[6-(benzylamino)pyrimidin-4-yl]-3-hydroxypiperidin-3-yl]methyl]-4-[(4,4-dimethylpiperidin-1-yl)methyl]-2-hydroxybenzamide | 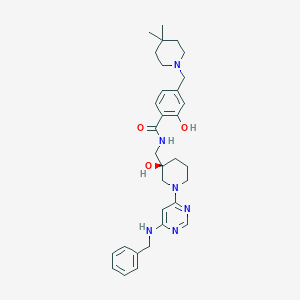 | ^213, 214^ |
| Quercetin | Non-SAM analogs | 2-(3,4-dihydroxyphenyl)-3,5,7-trihydroxychromen-4-one | 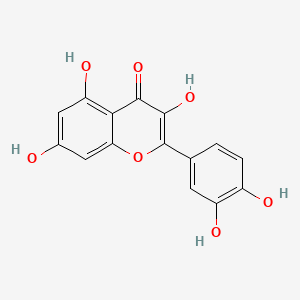 | ^215^ |
| CDIBA | Mutagenic inhibitor | 4-[2-(1-benzhydryl-5-chloro-2-methylindol-3-yl)ethoxy]benzoic acid | 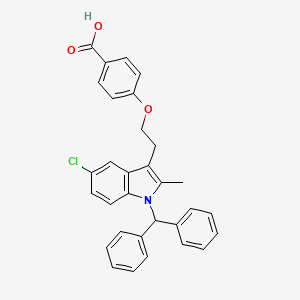 | ^213, 214^ |
| Eltrombopag | Mutagenic inhibitor | 3-[3-[[2-(3,4-dimethylphenyl)-5-methyl-3-oxo-1H-pyrazol-4-yl]diazenyl]-2-hydroxyphenyl]benzoic acid | 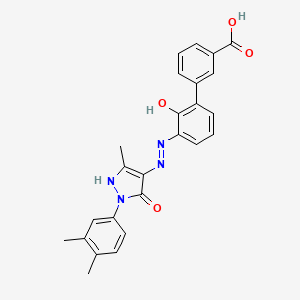 | ^213, 214^ |
| Hesperidin | Chinese Medicine Active Ingredient | (2S)-5-hydroxy-2-(3-hydroxy-4-methoxyphenyl)-7-[(2S,3R,4S,5S,6R)-3,4,5-trihydroxy-6-[[(2R,3R,4R,5R,6S)-3,4,5-trihydroxy-6-methyloxan-2-yl]oxymethyl]oxan-2-yl]oxy-2,3-dihydrochromen-4-one | 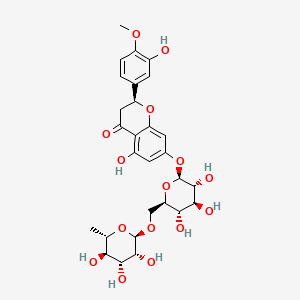 | ^183^ |
